# Probing the Interactions of Thiazole Abietane Inhibitors with the Human Serine Hydrolases ABHD16A and ABHD12

**DOI:** 10.1021/acsmedchemlett.3c00313

**Published:** 2023-09-18

**Authors:** Tiina J. Ahonen, Choa P. Ng, Beatriz Farinha, Bárbara Almeida, Bruno L. Victor, Christopher Reynolds, Eija Kalso, Jari Yli-Kauhaluoma, Jennifer Greaves, Vânia M. Moreira

**Affiliations:** † Drug Research Program, Division of Pharmaceutical Chemistry and Technology, Faculty of Pharmacy, University of Helsinki, 00014 Helsinki, Finland; ‡ Research Centre for Health and Life Sciences, Coventry University, CV1 5RW Coventry, U.K.; § BioISI—Biosystems & Integrative Sciences Institute, Faculty of Sciences, University of Lisbon, 1749-016 Lisboa, Portugal; ∥ School of Life Sciences, University of Essex, CO4 3SQ Colchester, U.K.; ⊥ Department of Pharmacology, Faculty of Medicine, University of Helsinki, 00014 Helsinki, Finland; # Department of Anaesthesiology, Intensive Care and Pain Medicine, Helsinki University Hospital and University of Helsinki, FI-00029 Helsinki, Finland; ¶ Centre for Neuroscience and Cell Biology, and Centre for Innovative Biomedicine and Biotechnology, University of Coimbra, 3000-548 Coimbra, Portugal; ∇ Laboratory of Pharmaceutical Chemistry, Faculty of Pharmacy, University of Coimbra, 3000-548 Coimbra, Portugal

**Keywords:** ABHD16A, ABHD12, serine hydrolase, dehydroabietic acid, competitive ABPP

## Abstract

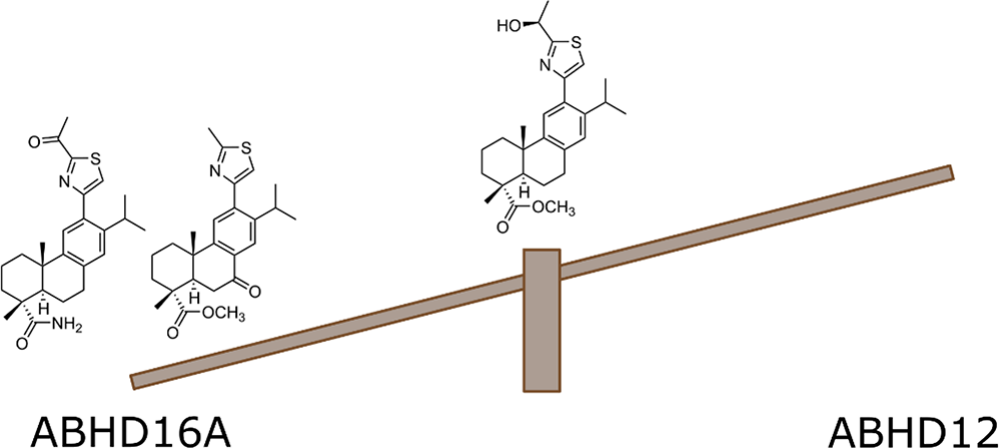

12-Thiazole abietanes are highly selective reversible inhibitors of hABHD16A that could potentially alleviate neuroinflammation. In this study, we used synthetic chemistry, competitive activity-based protein profiling, and computational methodologies to try to establish relevant structural determinants of activity and selectivity of this class of compounds for inhibiting ABHD16A over ABHD12. Five compounds significantly inhibited hABHD16A but also very efficiently discriminated between inhibition of hABHD16A and hABHD12, with compound **35** being the most effective, at 100 μM (55.1 ± 8.7%; *p* < 0.0001). However, an outstanding switch in the selectivity toward ABHD12 was observed in the presence of a ring A ester, if the C2′ position of the thiazole ring possessed a 1-hydroxyethyl group, as in compound **28**. Although our data were inconclusive as to whether the observed enzyme inhibition is allosteric or not, we anticipate that the structure–activity relationships presented herein will inspire future drug discovery efforts in this field.

The metabolic serine hydrolases ABHD12 and ABHD16A belong to the α,β-hydrolase domain (ABHD) family of enzymes, with important roles in lipid signaling and metabolism.^1^ ABHD12 is mainly expressed in macrophages and microglia and throughout the brain.^1,2^ ABHD16A is highly expressed in the brain, testis, muscle, and heart.^1,3^ It has also been identified in human platelet and mouse megakaryocyte membranes and extracellular vesicles derived from colorectal cancer cells.^4,5^ Both enzymes exist in the endoplasmic reticulum membrane and regulate the levels of signaling lipids in a concerted way.^6−9^ ABHD16A converts PS to lyso-PS, whereas ABHD12 hydrolyzes lyso-PS to glycerophosphoserine.^10^ Lyso-PS has several functions related to the immune response.^11−15^ Genetic deletion of ABHD12 leads to accumulation of lyso-PS in mice brain, leading to the neurodegenerative syndrome resembling the human neurological disorder PHARC (polyneuropathy, hearing loss, ataxia, retinitis pigmentosa, and cataract).^8,16^ ABHD16A polymorphism is associated with Kawasaki disease,^17^ and total loss of function of ABHD16A has been detected in patients with complicated hereditary spastic paraplegia.^18^ Increased expression of ABHD16A has also been linked to the promotion of gastric cancer metastasis.^19^

ABHD12 and ABHD16A possess a catalytic triad of Ser-His-Asp which functions through a well-established canonical esterase mechanism.^20,21^ However, the lack of available crystal structures of the two enzymes has hampered drug discovery, and selectivity toward the inhibition of ABHD16A among other serine hydrolases, including ABHD12, remains a challenge. Whereas inhibiting ABHD16A could be beneficial to alleviate the conditions associated with elevated lyso-PS levels, concomitant inhibition of ABHD12 would lead to undesirable effects.

Although there has been progress in understanding the *in vivo* role of ABHD12, in recent years, with the discovery of selective inhibitors,^22−24^ knowledge of the human ABHD16A (hABHD16A) is still unexplored. The general lipase inhibitors (−)-tetrahydrolipstatin (THL, **1**) and methyl arachidonyl fluorophosphonate (MAFP, **3**) also inhibit ABHD12 and ABHD16A (Figure 1).^21,25,28^ Reported ABHD12 inhibitors include triterpenoids **4**–**6** (Figure 1) with low micromolar IC_50_ values and unprecedented selectivity for ABHD12.^24^ Other ABHD12 inhibitors include *N*-3-pyridyl-*N′*-4-piperidinylthiourea derivatives (e.g., DO264, compound **7**, Figure 1)^23,27^ and urea analogs (e.g., **8**, Figure 1).^24^ Potent ABHD16A inhibitors, with a certain degree of selectivity, include 1,3,4-oxadiazol-2(3*H*)-ones (e.g., **12**, Figure 1)^25^ and α-alkylidene-β-lactone-based inhibitors (e.g., **11**, Figure 1), yet were not suitable for *in vivo* studies.^10^ Finally, 12-thiazole abietanes were reported as reversible inhibitors of human ABHD16A (Figure 1, compounds **13** and **14**), some with outstanding selectivity among a panel of other serine hydrolyses from rat cerebellar membrane proteome.^26^

**Figure 1:**
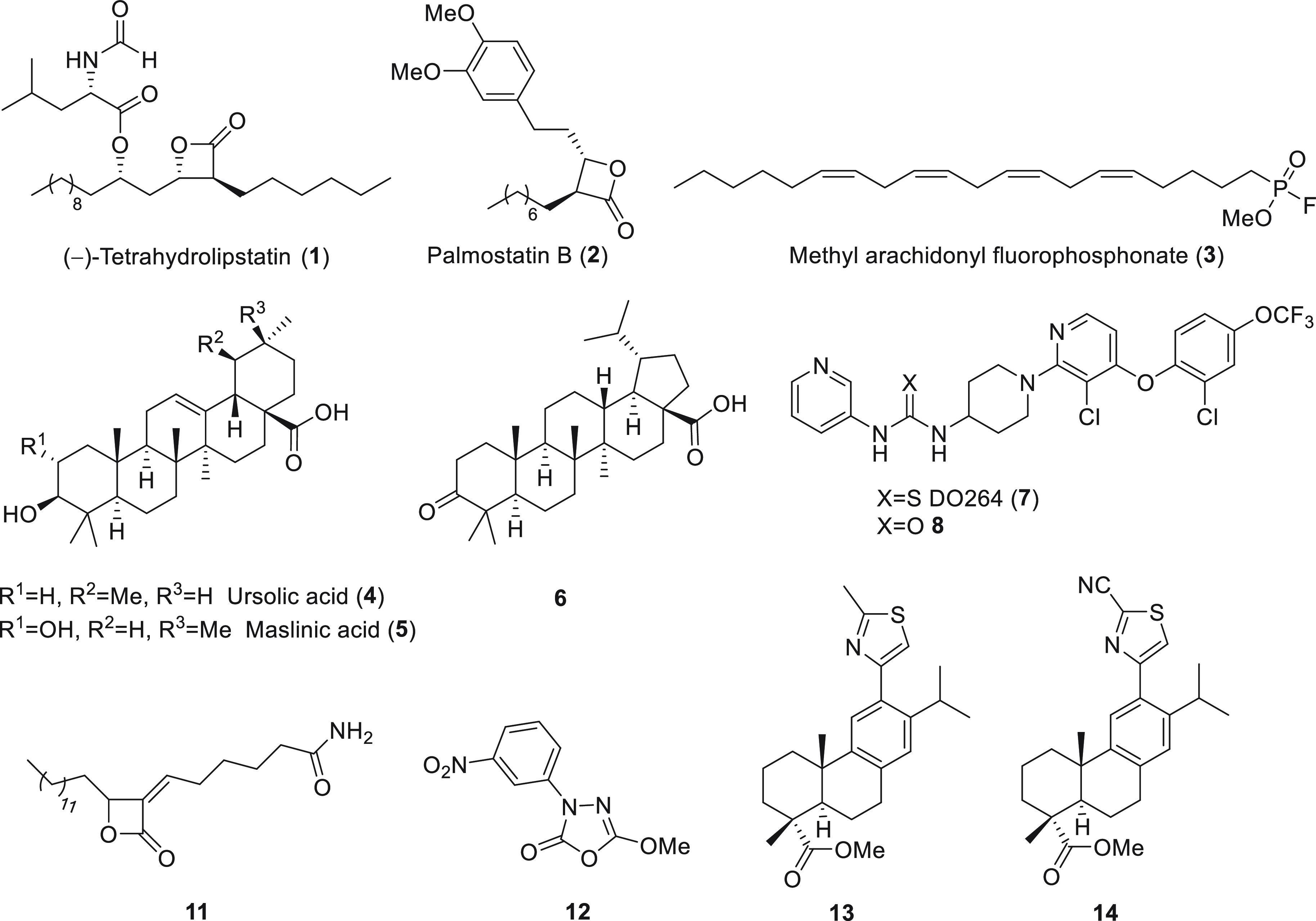
ABHD16A and ABHD12 inhibitors.

In this work, we set out to establish general chemical determinants of compound selectivity for ABHD16A over ABHD12, using 12-thiazole abietanes as a study model. Through synthetic chemistry, we designed novel compound sets to build robust structure–activity. Enzyme inhibition studies were made through competitive activity-based protein profiling (cABPP), a chemical proteomic method that measures the binding of reactive probes to the active site of an enzyme, such as fluorophosphonate (FP) probes which specifically and irreversibly target the reactive serine of serine hydrolases.^29,30^ Compounds that bind and inhibit their target enzyme interfere with reactive probe binding, enabling small molecules to be efficiently screened for potency and selectivity. We used an azido-FP probe that we conjugated to an infrared dye by azide–alkyne cycloaddition as an activity-based fluorescent reporter to quantitatively measure changes in the activity of recombinantly expressed ABHD12 and ABHD16A with the synthesized compounds which we detected by fluorescent Western blotting. Finally, computational models available from AlphaFold were used to explore the interaction of our sets of compounds with the active sites of each enzyme.

To learn how restrictive the presence of a substituent on ring A is, it was abrogated by conversion into a free methyl group (Scheme 1, part I). This was achieved from compound **19**, synthesized from dehydroabietic acid (**15**) via reduction of the ring A carboxyl group, followed by tosylation to give **16**, which was then reduced to **17**.^31^ Friedel–Crafts acylation of **17** gave **18**, and subsequent bromination gave **19**, as described before,^26^ with **19** isolated as a mixture of mono- and dibrominated compounds. The thiazole, 2-methylthiazole, and 2-aminothiazole derivatives **20**–**22** were prepared from **19**. In addition, compounds were designed with ketone, ester, and hydroxyl groups on the thiazole ring to probe how hydrogen bonding might affect the activity of the abietane inhibitors (Scheme 1, part II). In this set, compounds **23**, **24**, and **19** were reacted with (2*S*)-2-(acetyloxy)propanethioamide (**S38**, Supporting Information, Scheme S1)^32,33^ to give the respective thiazoles **25**–**27** (Scheme 1, part II). Deacetylation of **25**–**27** in aqueous sodium hydroxide solution gave compounds **28**–**30**, which were oxidized with Dess–Martin periodinane to give the final compounds **31**–**33** (Scheme 1, part II).

**Scheme 1:**
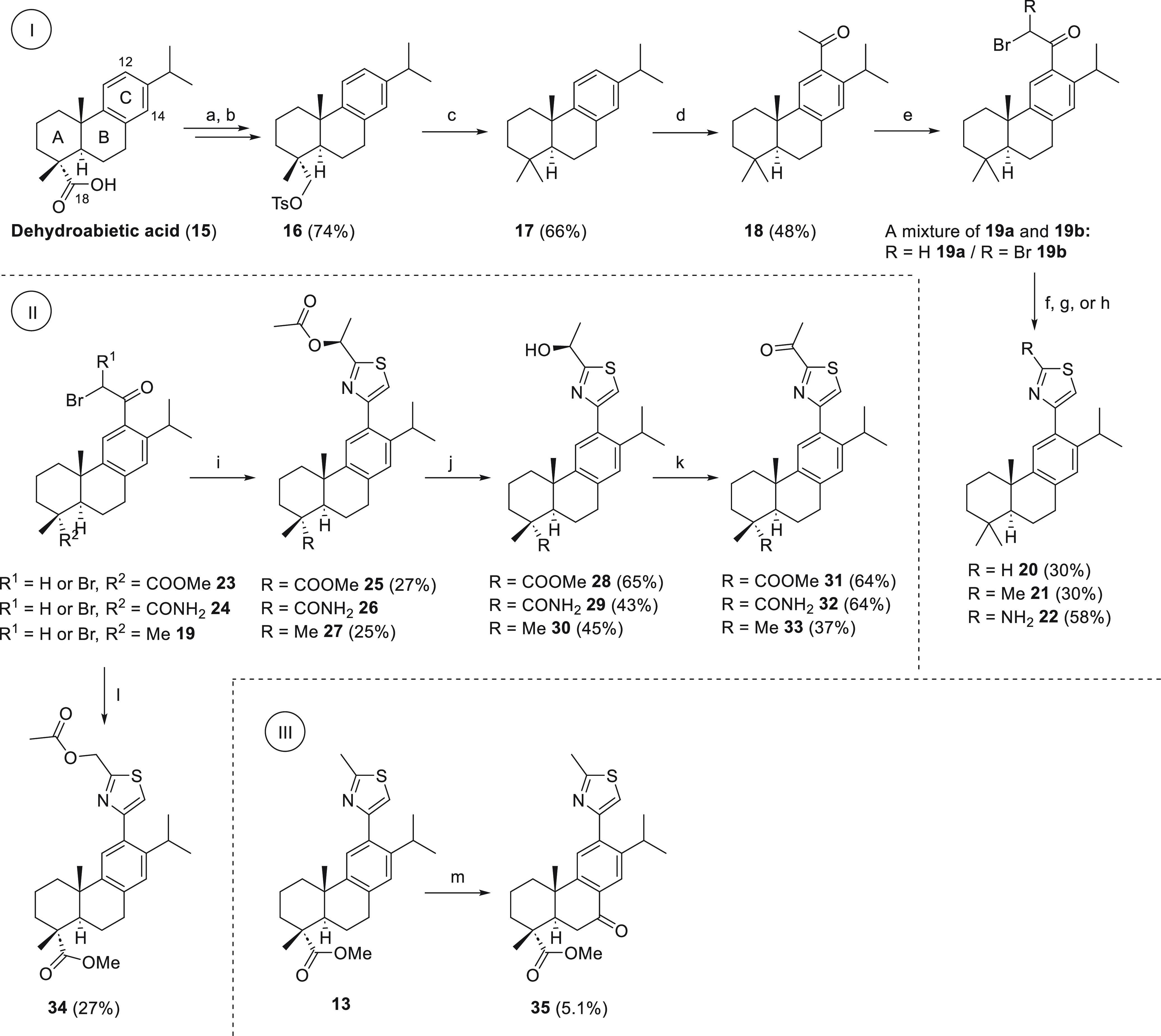
Synthesis of 12-Thiazole Abietanes Reagents and conditions: (a) LiAlH_4_, THF, 0 °C → rt, 3 h; (b) *p*-toluenesulfonyl chloride, pyridine, 0 °C → rt, 24 h; (c) NaI, Zn (powder), DMF, 100 °C, 7 d; (d) MeCOCl, AlCl_3_, CH_2_Cl_2_, 0 °C → rt, 3.5 h; (e) CuBr_2_, MeOH, 65 °C, 16 h; (f) thioformamide, dry 1,4-dioxane, 100 °C with microwaves, 10 min, yield over 2 steps; (g) thioacetamide, dry EtOH, 120 °C with microwaves, 30 min, yield over 2 steps; (h) thiourea, dry EtOH, 120 °C with microwaves, 2 h, yield over 2 steps; (i) (2*S*)-2-(acetyloxy)propanethioamide (**S38**), dry EtOH, 120 °C with microwaves, 30 min, yields over two steps; (j) NaOH (aq), MeOH, rt, 2 h to 5 d; (k) Dess–Martin periodinane, CH_2_Cl_2_, rt, 3–26 h; (l) 2-(acetyloxy)ethanethioamide (**S41**) dry EtOH, 120 °C with microwaves, 30 min; (m) *tert*-butyl hydroperoxide, NaClO_2_, acetonitrile, H_2_O, ethyl acetate, 60 °C, 7 d. The yields are reported after chromatographic purification.

The synthesis of **34** from **19** and 2-(acetyloxy)ethanethioamide, prepared from glycolamide, was also accomplished (S41, Supporting Information, Scheme 1). Finally, the keto derivative **35** was made from **23**, via the 2′-methylthiazole **13**,^26^ followed by benzylic oxidation (Scheme 1, part III).^34^

Through cABPP on both murine (mABHD16A) and hABHD16A enzymes, we found that only five significantly inhibited (≥50% inhibition; *p* ≤ 0.05) mABHD16A activity, at 200 μM, whereas the activity of the enzyme was almost completely abrogated by palmostatin B, at 100 μM (94.7 ± 1.5%; *p* < 0.0001) (Supporting Information, Figure S1). These included **28** (66.4 ± 4.7%; *p* = 0.003); **29** (59.8 ± 8.3%; *p* = 0.01); **32** (83.5 ± 5.6%; *p* < 0.0001); **34** (62.9 ± 2.0%; *p* = 0.007); **35** (73.2 ± 5.5%; *p* = 0.0007). Additionally, **32** at a lower concentration of 20 μM significantly inhibited mABHD16A activity (55.5 ± 15.9%; *p* = 0.03). The same five compounds also inhibited the human enzyme significantly, at a lower concentration of 100 μM (Figure 2). Compound **28** inhibited hABHD16A activity by 48.0 ± 5.7% (*p* = 0.0007), **29** by 37.6 ± 11.7% (*p* = 0.02), and **32** by 45.5 ± 4.7% (*p* = 0.001). Compound **34** inhibited hABHD16A activity by 48.4 ± 3.1% (*p* = 0.0006). Compound **35** was the most effective inhibitor of hABHD16A (55.1 ± 8.7%; *p* < 0.0001). Notably, compounds **28**, **32**, and **35** also significantly inhibited hABHD16A activity at 20 μM (**28** inhibited by 35.7 ± 11.0% (*p* = 0.02), **32** inhibited by 33.5 ± 7.6% (*p* = 0.03), and **35** inhibited by 32.2 ± 6.0% (*p* = 0.04)).

**Figure 2:**
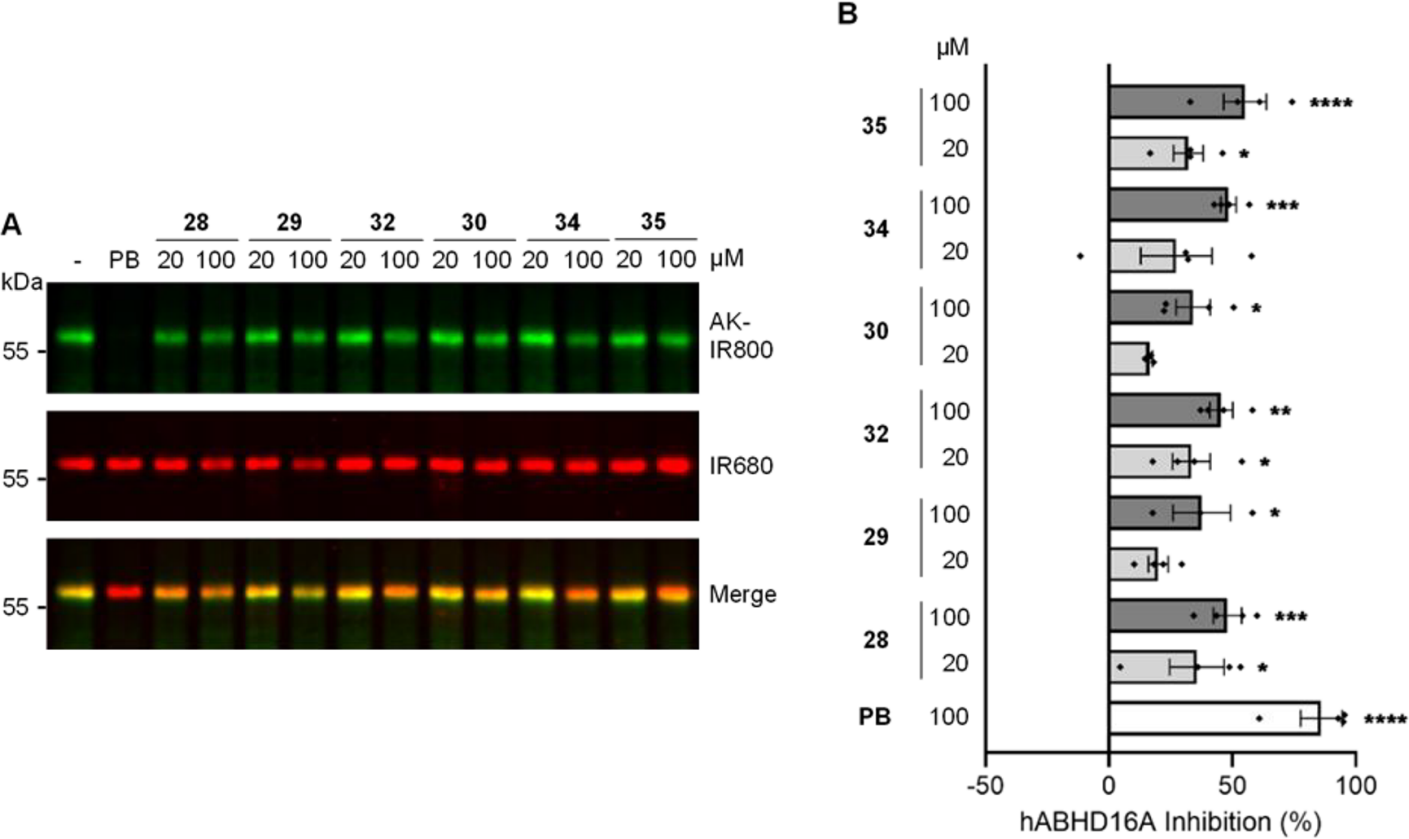
Inhibition of hABHD16A by cABPP. HA-tagged hABHD16A enriched from HEK293T total membrane proteomes was incubated with the compounds followed by labeling with FP-azide and conjugation by click chemistry to an alkyne-infrared 800 dye (AK-IR800). Anti-HA primary antibody and an anti-rat IR680 secondary antibody were used to detect hABHD16A (IR680). hABHD16A inhibition was calculated by measuring the difference in FP-azide incorporation relative to DMSO control (−), normalized to protein levels. 100 μM palmostatin B (PB) was used as a positive control. (A) Representative immunoblot images show click chemistry signal (top, AK-IR800), HA (middle, IR680), and merge (bottom). The position of molecular weight standards is shown on the left. (B) Bar chart showing mean percentage hABHD16A inhibition. Individual data points represent independent experiments. Error bars represent ±SEM. Statistical significance was determined by one-way ANOVA with Dunnett’s post hoc test. Only statistically significant analysis is shown: *p < 0.05; **p < 0.01; ***p < 0.001; ****p < 0.0001.

In contrast to hABHD16A, out of the five compounds tested, only **28** significantly inhibited hABHD12, as determined by cABPP (Figure 3). Compound **28** was effective at inhibiting hABHD12 at both 100 μM (60.9 ± 4.4%; *p* = 0.02) and 20 μM (54.0 ± 14.9%; *p* < 0.05). For comparison, 100 μM palmostatin B inhibited hABHD12 activity by 73.0 ± 10.5%; *p* = 0.003). For this reason, we decided to also test compound **30** for inhibition of both enzymes, as it shares a common 2′-(1-hydroxyethyl)thiazole substituent on ring C of the abietane with **28** and **29**, yet they all possess different functional groups attached onto ring A. The activity of compound **30** was similar to **29**, as it inhibited hABHD16A by 34.1 ± 6.9% (*p* = 0.03) but not ABHD12. There was a small increase in hABHD12 FP-azide labeling with compounds **30** and **32**, which may reflect altered rates of azido-FP reactivity with the active site of ABHD12; however, when analyzed by one-way ANOVA, this was not found to be statistically significant.

**Figure 3:**
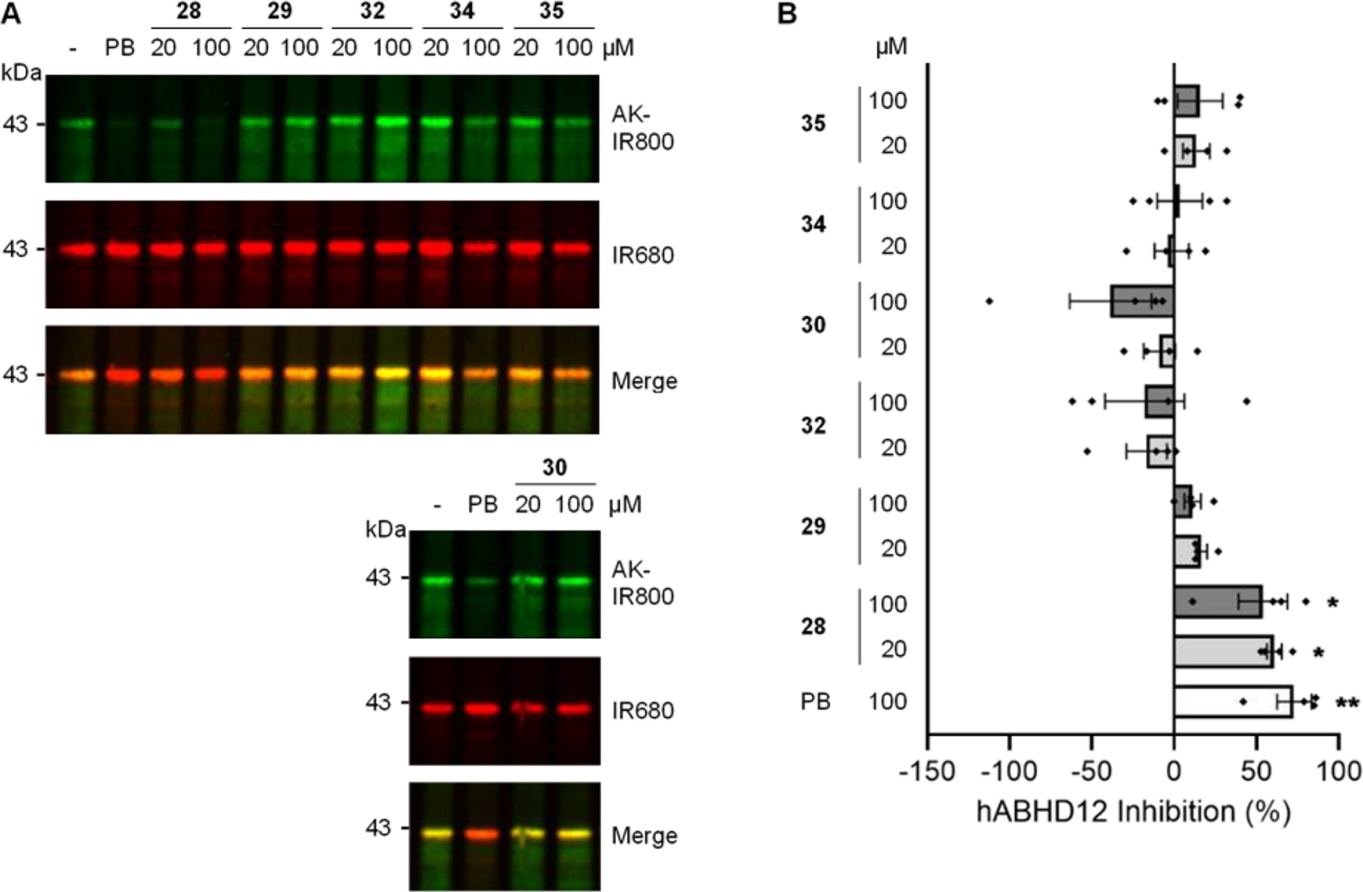
Inhibition of hABHD12 by cABPP. HA-tagged hABHD12 enriched from HEK293T total membrane proteomes was incubated with the compounds followed by labeling with FP-azide and conjugation by click chemistry to an alkyne-infrared 800 dye (AK-IR800). Anti-HA primary antibody and an anti-rat IR680 secondary antibody were used to detect hABHD12 (IR680). hABHD12 inhibition was calculated by measuring the difference in FP-azide incorporation relative to DMSO control (−), normalized to protein levels. 100 μM palmostatin B (PB) was used as a positive control. (A) Representative immunoblot images are shown: click chemistry signal (top, AK-IR800), HA (middle, IR680), and merge (bottom). The position of molecular weight standards is shown on the left. (B) Bar chart showing mean percentage hABHD12 inhibition. Individual data points represent independent experiments. Error bars represent ± SEM. Statistical significance was determined by one-way ANOVA with Dunnett’s post hoc test. Only statistically significant analysis is shown.: *p < 0.05; **p < 0.01.

A look at how promiscuous **28** binds on the AlphaFold models showed the thiazole ring positioned toward the catalytic Ser residue instead of the ester group on ring A, in both enzymes (Figure 4A,B). We should note that the ester group on ring A of the abietanes is exceptionally stable and only affected by harsh reactions conditions, such as strong base.^26,35−37^ Whereas in ABHD12, the hydroxyl group of the thiazole ring of **28** is hydrogen bonded with the catalytic Ser residue (Figure 4A), in ABHD16A, the same group is hydrogen bonded to a carbonyl group belonging to Ile 417 (Figure 4B). Such differences do not translate in significant changes in the docking poses or in the calculated binding free energy, which is consistent with the fact that **28** inhibits both enzymes. Molecular docking with **35** also showed similar poses on both proteins (Figure 4E,F). However, the ester of **35** is hydrogen bonded to Gln 97 in ABHD12 and to Thr 387 in ABHD16A (Figure 4E,F). Nonetheless, the determined binding free energy difference obtained for this compound on both protein models is within the error of the method and consequently insufficient to explain the selectivity for ABHD16A. Finally, molecular docking of **13** (Figure 4C,D), also a selective inhibitor of ABHD16A and yet devoid of the carbonyl group on ring B, was reassuring of a consistent binding mode in our model for structurally similar compounds.

**Figure 4:**
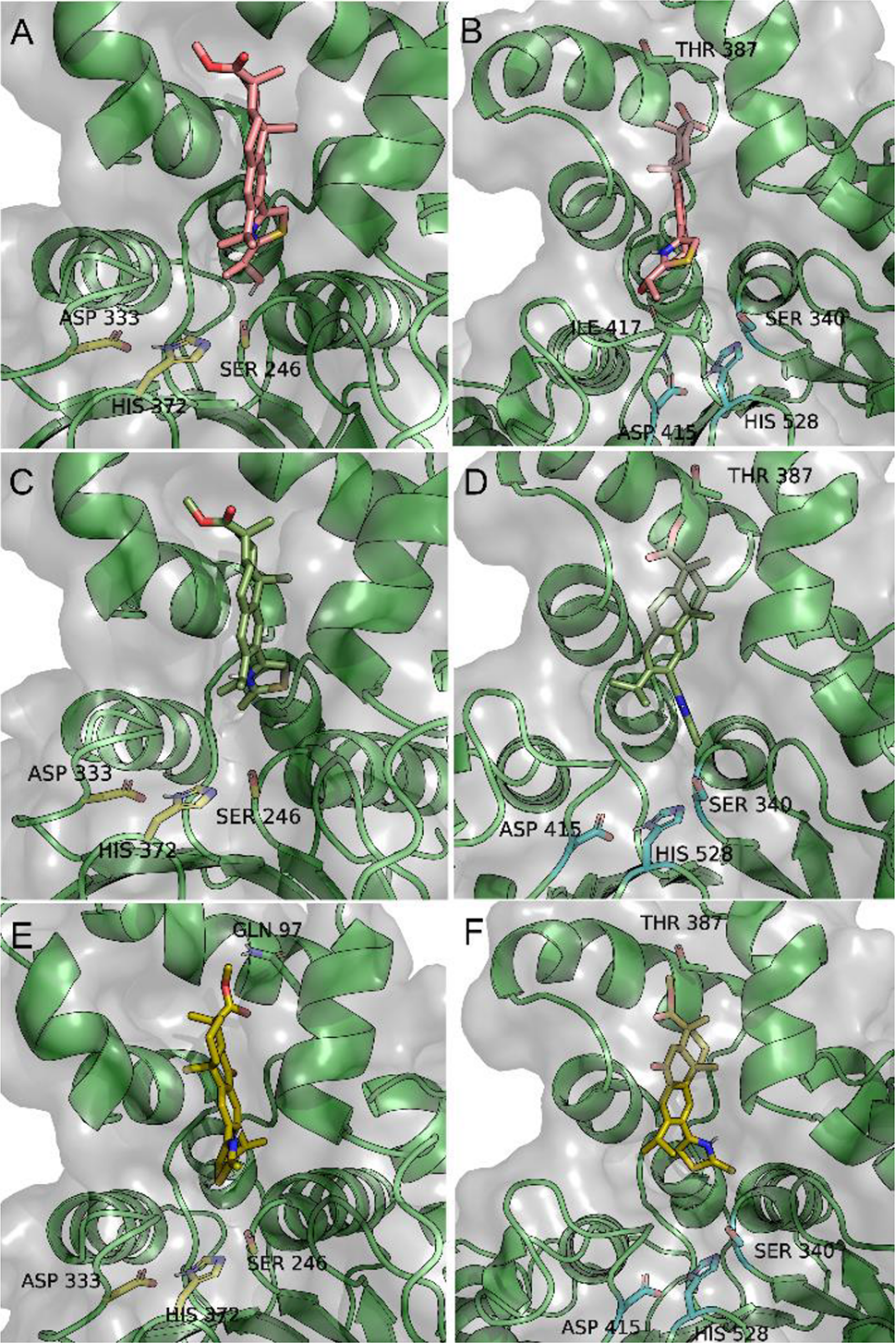
Docking solution for **28** (pink), **13** (green), and **35** (yellow) on the binding sites of ABHD12 (A, C, E) and ABHD16A (B, D, F), close to the catalytic triad. The side chains of the catalytic triad residues are in yellow (ABHD12) and cyan (ABHD16A) sticks.

With this work we show that (1) a thiazole on ring C accompanied by a convenient substituent on ring A is a key feature for the inhibitory activity of these compounds; (2) ring A functional groups are somewhat permissible and include at least ester, amide, and hydroxyl, depending on the pattern of substitution at the C2′ position of the thiazole, attached to ring C; (3) substituents capable of hydrogen bonding at the C2′ position of the thiazole ring are best when compared to bulky apolar substituents for inhibition of ABHD16A; and (4) minor modifications such as the introduction of a carbonyl group on ring B do not significantly affect the activity of the inhibitors.

Overall, compounds with these general features are selective toward ABHD16A. There is, however, an outstanding switch in the selectivity behavior toward ABHD12, which occurs only in the presence of a ring A ester, if the C2′ position of the thiazole ring possesses a 1-hydroxyethyl group, as in **28**. Despite our efforts, the AlphaFold models do not explain inhibitor selectivity for our compound series. Interactions at the binding site were addressed, yet we cannot rule out the possibility of allosteric inhibition, an issue that will require probing alternative binding sites on the surface of the proteins. Finally, it will also be interesting to test the neuroprotective effects of these inhibitors, for instance in lipopolysaccharide-induced neurodegeneration models in neuron-glia cell cultures or human induced pluripotent stem cells. Such aspects will be the basis of future studies in this field.

## Supporting Information Available

Synthetic procedures, analytical data, assay protocols, and computational procedures (PDF)

## Abbreviations

ABHD: α,β-hydrolase domain
ABPP: activity-based protein profiling
DMF: *N,N*-dimethylformamide
DMSO: dimethyl sulfoxide
FP: fluorophosphonate
lyso-PS: lysophosphatidylserine
MAFP: methyl arachidonyl fluorophosphonate
PS: phosphatidylserine
THF: tetrahydrofuran
THL: (−)-tetrahydrolipstatin

## References

[ref1] Lord C. C.; Thomas G.; Brown J. M. Mammalian Alpha Beta Hydrolase Domain (ABHD) Proteins: Lipid Metabolizing Enzymes at the Interface of Cell Signaling and Energy Metabolism. Biochim. Biophys. Acta, Mol. Cell Biol. Lipids 2013, 1831 (4), 792–802. 10.1016/j.bbalip.2013.01.002. PMC4765316 23328280

[ref2] Wu C.; Jin X.; Tsueng G.; Afrasiabi C.; Su A. I. BioGPS: Building Your Own Mash-up of Gene Annotations and Expression Profiles. Nucleic Acids Res. 2016, 44 (D1), D313–D316. 10.1093/nar/gkv1104. 26578587 PMC4702805

[ref3] Turcotte C.; Dumais É.; Archambault A.-S.; Martin C.; Blanchet M.-R.; Bissonnette É.; Boulet L.-P.; Laviolette M.; Di Marzo V.; Flamand N. Human Leukocytes Differentially Express Endocannabinoid-Glycerol Lipases and Hydrolyze 2-Arachidonoyl-Glycerol and Its Metabolites from the 15-Lipoxygenase and Cyclooxygenase Pathways. J. Leukocyte Biol. 2019, 106, 1337–1347. 10.1002/JLB.3A0919-049RRR. 31556464

[ref4] Senis Y. A.; Tomlinson M. G.; García Á.; Dumon S.; Heath V. L.; Herbert J.; Cobbold S. P.; Spalton J. C.; Ayman S.; Antrobus R.; Zitzmann N.; Bicknell R.; Frampton J.; Authi K. S.; Martin A.; Wakelam M. J. O.; Watson S. P. A Comprehensive Proteomics and Genomics Analysis Reveals Novel Transmembrane Proteins in Human Platelets and Mouse Megakaryocytes Including G6b-B, a Novel Immunoreceptor Tyrosine-Based Inhibitory Motif Protein. Mol. Cell. Proteomics 2007, 6 (3), 548–564. 10.1074/mcp.D600007-MCP200. 17186946 PMC1860054

[ref5] Hong B. S.; Cho J.-H.; Kim H.; Choi E.-J.; Rho S.; Kim J.; Kim J. H.; Choi D.-S.; Kim Y.-K.; Hwang D.; Gho Y. S. Colorectal Cancer Cell-Derived Microvesicles Are Enriched in Cell Cycle-Related MRNAs That Promote Proliferation of Endothelial Cells. BMC Genomics 2009, 10 (1), 556 10.1186/1471-2164-10-556. 19930720 PMC2788585

[ref6] Joshi A.; Shaikh M.; Singh S.; Rajendran A.; Mhetre A.; Kamat S. S. Biochemical Characterization of the PHARC-Associated Serine Hydrolase ABHD12 Reveals Its Preference for Very-Long-Chain Lipids. J. Biol. Chem. 2018, 293 (44), 16953–16963. 10.1074/jbc.RA118.005640. 30237167 PMC6217928

[ref7] Singh S.; Joshi A.; Kamat S. S. Mapping the Neuroanatomy of ABHD16A, ABHD12, and Lysophosphatidylserines Provides New Insights into the Pathophysiology of the Human Neurological Disorder PHARC. Biochemistry 2020, 59 (24), 2299–2311. 10.1021/acs.biochem.0c00349. 32462874 PMC7311203

[ref8] Blankman J. L.; Long J. Z.; Trauger S. A.; Siuzdak G.; Cravatt B. F. ABHD12 Controls Brain Lysophosphatidylserine Pathways That Are Deregulated in a Murine Model of the Neurodegenerative Disease PHARC. Proc. Natl. Acad. Sci. U. S. A. 2013, 110 (4), 1500–1505. 10.1073/pnas.1217121110. 23297193 PMC3557017

[ref9] Nguyen T. T.; Voeltz G. K. An ER Phospholipid Hydrolase Drives ER-Associated Mitochondrial Constriction for Fission and Fusion. eLife 2022, 11, e84279 10.7554/eLife.84279. 36448541 PMC9725753

[ref10] Kamat S. S.; Camara K.; Parsons W. H.; Chen D. H.; Dix M. M.; Bird T. D.; Howell A. R.; Cravatt B. F. Immunomodulatory Lysophosphatidylserines Are Regulated by ABHD16A and ABHD12 Interplay. Nat. Chem. Biol. 2015, 11 (2), 164–171. 10.1038/nchembio.1721. 25580854 PMC4301979

[ref11] Martin T. W.; Lagunoff D. Interactions of Lysophospholipids and Mast Cells. Nature 1979, 279 (5710), 250–252. 10.1038/279250a0. 86956

[ref12] Bruni A.; Bigon E.; Battistella A.; Boarato E.; Mietto L.; Toffano G. Lysophosphatidylserine as Histamine Releaser in Mice and Rats. Agents Actions 1984, 14 (5–6), 619–625. 10.1007/BF01978896. 6206697

[ref13] Frasch S. C.; Berry K. Z.; Fernandez-Boyanapalli R.; Jin H.-S.; Leslie C.; Henson P. M.; Murphy R. C.; Bratton D. L. NADPH Oxidase-Dependent Generation of Lysophosphatidylserine Enhances Clearance of Activated and Dying Neutrophils via G2A. J. Biol. Chem. 2008, 283 (48), 33736–33749. 10.1074/jbc.M807047200. 18824544 PMC2586279

[ref14] Xu Y.; Casey G.; Mills G. B. Effect of Lysophospholipids on Signaling in the Human Jurkat T Cell Line. J. Cell. Physiol. 1995, 163 (3), 441–450. 10.1002/jcp.1041630303. 7775587

[ref15] Barnes M. J.; Cyster J. G. Lysophosphatidylserine Suppression of T-Cell Activation via GPR174 Requires Gαs Proteins. Immunol. Cell Biol. 2018, 96 (4), 439–445. 10.1111/imcb.12025. 29457279 PMC5916342

[ref16] Fiskerstrand T.; H’mida-Ben Brahim D.; Johansson S.; M’zahem A.; Haukanes B. I.; Drouot N.; Zimmermann J.; Cole A. J.; Vedeler C.; Bredrup C.; Assoum M.; Tazir M.; Klockgether T.; Hamri A.; Steen V. M.; Boman H.; Bindoff L. A.; Koenig M.; Knappskog P. M. Mutations in ABHD12 Cause the Neurodegenerative Disease PHARC: An Inborn Error of Endocannabinoid Metabolism. Am. J. Hum. Genet. 2010, 87 (3), 410–417. 10.1016/j.ajhg.2010.08.002. 20797687 PMC2933347

[ref17] Hsieh Y.-Y.; Lin Y.-J.; Chang C.-C.; Chen D.-Y.; Hsu C.-M.; Lo M.-M.; Hsu K.-H.; Tsai F.-J. Human Lymphocyte Antigen B-Associated Transcript 2, 3, and 5 Polymorphisms and Haplotypes Are Associated with Susceptibility of Kawasaki Disease and Coronary Artery Aneurysm. J. Clin. Lab. Anal. 2010, 24 (4), 262–268. 10.1002/jcla.20409. 20626023 PMC6647560

[ref18] Yahia A.; Elsayed L. E. O.; Valter R.; Hamed A. A. A.; Mohammed I. N.; Elseed M. A.; Salih M. A.; Esteves T.; Auger N.; Abubaker R.; Koko M.; Abozar F.; Malik H.; Adil R.; Emad S.; Musallam M. A.; Idris R.; Eltazi I. Z. M.; Babai A.; Ahmed E. A. A.; Abd Allah A. S. I.; Mairey M.; Ahmed A. K. M. A.; Elbashir M. I.; Brice A.; Ibrahim M. E.; Ahmed A. E.; Lamari F.; Stevanin G. Pathogenic Variants in ABHD16A Cause a Novel Psychomotor Developmental Disorder With Spastic Paraplegia. Front. Neurol. 2021, 12, 720201 10.3389/fneur.2021.720201. 34489854 PMC8417901

[ref19] Yang L.; Hou Y.; Du Y.; Li Q.; Zhou F.; Li Y.; Zeng H.; Jin T.; Wan X.; Guan S.; Wang R.; Liu M. Mirtronic MiR-4646-5p Promotes Gastric Cancer Metastasis by Regulating ABHD16A and Metabolite Lysophosphatidylserines. Cell Death Differ. 2021, 28, 2708 10.1038/s41418-021-00779-y. 33875796 PMC8408170

[ref20] Rauwerdink A.; Kazlauskas R. J. How the Same Core Catalytic Machinery Catalyzes 17 Different Reactions: The Serine-Histidine-Aspartate Catalytic Triad of α/β-Hydrolase Fold Enzymes. ACS Catal. 2015, 5 (10), 6153–6176. 10.1021/acscatal.5b01539. 28580193 PMC5455348

[ref21] Navia-Paldanius D.; Savinainen J. R.; Laitinen J. T. Biochemical and Pharmacological Characterization of Human α/β-Hydrolase Domain Containing 6 (ABHD6) and 12 (ABHD12). J. Lipid Res. 2012, 53 (11), 2413–2424. 10.1194/jlr.M030411. 22969151 PMC3466009

[ref22] Parkkari T.; Haavikko R.; Laitinen T.; Navia-Paldanius D.; Rytilahti R.; Vaara M.; Lehtonen M.; Alakurtti S.; Yli-Kauhaluoma J.; Nevalainen T.; Savinainen J. R.; Laitinen J. T. Discovery of Triterpenoids as Reversible Inhibitors of α/β-Hydrolase Domain Containing 12 (ABHD12). PLoS One 2014, 9 (5), e98286 10.1371/journal.pone.0098286. 24879289 PMC4045134

[ref23] Ogasawara D.; Ichu T.-A.; Vartabedian V. F.; Benthuysen J.; Jing H.; Reed A.; Ulanovskaya O. A.; Hulce J. J.; Roberts A.; Brown S.; Rosen H.; Teijaro J. R.; Cravatt B. F. Selective Blockade of the Lyso-PS Lipase ABHD12 Stimulates Immune Responses in Vivo. Nat. Chem. Biol. 2018, 14 (12), 1099–1108. 10.1038/s41589-018-0155-8. 30420694 PMC6263940

[ref24] Grice C. A.; Moody J. V.; Buzard D. J.; Cisar J. S. ABHD12 Inhibitors and Methods of Making and Using Same. WO/2020/232153, 2020.

[ref25] Savinainen J. R.; Patel J. Z.; Parkkari T.; Navia-Paldanius D.; Marjamaa J. J. T.; Laitinen T.; Nevalainen T.; Laitinen J. T. Biochemical and Pharmacological Characterization of the Human Lymphocyte Antigen B-Associated Transcript 5 (BAT5/ABHD16A). PLoS One 2014, 9 (10), e109869 10.1371/journal.pone.0109869. 25290914 PMC4188605

[ref26] Ahonen T. J.; Savinainen J. R.; Yli-Kauhaluoma J. T.; Kalso E. A.; Laitinen J. T.; Moreira V. M. Discovery of 12-Thiazole Abietanes as Selective Inhibitors of the Human Metabolic Serine Hydrolase HABHD16A. ACS Med. Chem. Lett. 2018, 9 (12), 1269–1273. 10.1021/acsmedchemlett.8b00442. 30613338 PMC6296171

[ref27] Ogasawara D.; Ichu T.-A.; Jing H.; Hulce J. J.; Reed A.; Ulanovskaya O. A.; Cravatt B. F. Discovery and Optimization of Selective and in Vivo Active Inhibitors of the Lysophosphatidylserine Lipase α/β-Hydrolase Domain-Containing 12 (ABHD12). J. Med. Chem. 2019, 62 (3), 1643–1656. 10.1021/acs.jmedchem.8b01958. 30720278 PMC6583925

[ref28] Hoover H. S.; Blankman J. L.; Niessen S.; Cravatt B. F. Selectivity of Inhibitors of Endocannabinoid Biosynthesis Evaluated by Activity-Based Protein Profiling. Bioorg. Med. Chem. Lett. 2008, 18 (22), 5838–5841. 10.1016/j.bmcl.2008.06.091. 18657971 PMC2634297

[ref29] Liu Y.; Patricelli M. P.; Cravatt B. F. Activity-based protein profiling: the serine hydrolases. Proc. Natl. Acad. Sci. U. S. A. 1999, 96 (26), 14694–14699. 10.1073/pnas.96.26.14694. 10611275 PMC24710

[ref30] Faucher F.; Bennett J. M.; Bogyo M.; Lovell S. Strategies for Tuning the Selectivity of Chemical Probes that Target Serine Hydrolases. Cell Chem. Biol. 2020, 27 (8), 937–952. 10.1016/j.chembiol.2020.07.008. 32726586 PMC7484133

[ref31] Thommen C.; Jana C. K.; Neuburger M.; Gademann K. Syntheses of Taiwaniaquinone F and Taiwaniaquinol A via an Unusual Remote C-H Functionalization. Org. Lett. 2013, 15 (6), 1390–1393. 10.1021/ol4003652. 23461731

[ref32] Zhou J.; Liu P.; Chen S.; Wu Y.; Wang D.; Jia Z.; Qiao L.; Frietze W.; Xia M.; Dai Y. Processes of Preparing a JAK1 Inhibitor and New Forms Thereto. WO2015/168246, 2015.

[ref33] Schmidt U.; Gleich P.; Griesser H.; Utz R. Amino Acids and Peptides; 58 Synthesis of Optically Active 2-(1-Hydroxyalkyl)-thiazole-4-carboxylic Acids and 2-(1-Aminoalkyl)-thiazole-4-carboxylic Acids. Synthesis 1986, 1986 (12), 992–998. 10.1055/s-1986-31847.

[ref34] Kolsi L. E.; Krogerus S.; Brito V.; Rüffer T.; Lang H.; Yli-Kauhaluoma J.; Silvestre S. M.; Moreira V. M. Regioselective Benzylic Oxidation of Aromatic Abietanes: Application to the Semisynthesis of the Naturally Occurring Picealactones A, B and C. ChemistrySelect 2017, 2 (24), 7008–7012. 10.1002/slct.201701477.

[ref35] Kolsi L. E.; Leal A. S.; Yli-Kauhaluoma J.; Liby K. T.; Moreira V. M. Dehydroabietic oximes halt pancreatic cancer cell growth in the G1 phase through induction of p27 and downregulation of cyclin D1. Sci. Rep. 2018, 8, 15923 10.1038/s41598-018-34131-1. 30374056 PMC6206059

[ref36] González M. A.; Pérez-Guaita D.; Correa-Royero J.; Zapata B.; Agudelo L.; Mesa-Arango A.; Betancur-Galvis L. Eur. J. Med. Chem. 2010, 45, 811–816. 10.1016/j.ejmech.2009.10.010. 19892441

[ref37] Liu M.-L.; Pan X.-Y.; Yang T.; Zhang W.-M.; Wang T.-Q.; Wang H.-Y.; Lin H.-X.; Yang C.-G.; Cui Y.-M. Bioorg. Med. Chem. Lett. 2016, 26, 5492–5496. 10.1016/j.bmcl.2016.10.018. 27777007

