# Supplementary material for: Probing the Interactions of Thiazole Abietane Inhibitors with the Human Serine Hydrolases ABHD16A and ABHD12
Source: ACS Med Chem Lett. 2023 Sep 18;14(10):1404–10. doi: 10.1021/acsmedchemlett.3c00313 (PMC10577890; doi:10.1021/acsmedchemlett.3c00313)
Supplement: Supplementary file 1 — ml3c00313_si_001.pdf [file ml3c00313_si_001.pdf]

Probing the interactions of thiazole abietane inhibitors with the human serine hydrolases ABHD16A and ABHD12

Tiina J. Ahonen[†,#], Choa P. Ng[‡,#], Beatriz Farinha[§], Bárbara Almeida[§], Bruno L. Victor[§], Christopher Reynolds[‡,±], Eija Kalso[‖,ξ], Jari Yli-Kauhaluoma[†], Jennifer Greaves[‡,*], and Vânia M. Moreira[†,✝,⊥,*]

[†]Drug Research Program, Division of Pharmaceutical Chemistry and Technology, Faculty of Pharmacy, University of Helsinki, Finland

[‡]Research Centre for Health and Life Sciences, Coventry University, Coventry, UK

[§]BioISI - Biosystems & Integrative Sciences Institute, Faculty of Sciences, University of Lisbon, Lisboa, Portugal

[±]School of Life Sciences, University of Essex, Colchester, UK

[‖]Department of Pharmacology, Faculty of Medicine, University of Helsinki, Helsinki, Finland

[ξ]Department of Anaesthesiology, Intensive Care and Pain Medicine, Helsinki University Hospital and University of Helsinki, Finland

[✝]Centre for Neuroscience and Cell Biology, University of Coimbra, Portugal and Centre for Innovative Biomedicine and Biotechnology, University of Coimbra, Portugal

[⊥]Laboratory of Pharmaceutical Chemistry, Faculty of Pharmacy, University of Coimbra, Portugal.

[#] Equal contribution

Supporting information

Table of Contents

1. General chemistry methods S4
2. Synthesis S4
3. Recombinant expression of ABHD16A and ABHD12 in HEK293T cells S15
4. Competitive activity-based protein profiling (cABPP) in HEK293T membrane proteomes S15
5. Molecular modeling S17
6. NMR spectra S20

^{1}H NMR of compound **S38** in $CDCl_3$ S20
^{13}C NMR of compound **S38** in $CDCl_3$ S20
^{1}H NMR of compound **S41** in DMSO-d_6 .... S21
^{13}C NMR of compound **S41** in DMSO-d_6 .... S21
^{1}H NMR of compound **20** in $CDCl_3$ S22
^{13}C NMR of compound **20** in $CDCl_3$ S22
^{1}H NMR of compound **21** in $CDCl_3$ S23
^{13}C NMR of compound **21** in $CDCl_3$ S23
^{1}H NMR of compound **22** in $CDCl_3$ S24
^{13}C NMR of compound **22** in $CDCl_3$ S24
^{1}H NMR of compound **25** in $CDCl_3$ S25
^{13}C NMR of compound **25** in $CDCl_3$ S25
^{1}H NMR of compound **27** in $CDCl_3$ S26
^{13}C NMR of compound **27** in $CDCl_3$ S26
^{1}H NMR of compound **28** in $CDCl_3$ S27
^{13}C NMR of compound **28** in $CDCl_3$ S27
^{1}H NMR of compound **29** in $CDCl_3$ S28
^{13}C NMR of compound **29** in $CDCl_3$ S28
^{1}H NMR of compound **30** in $CDCl_3$ S29
^{13}C NMR of compound **30** in $CDCl_3$ S29
^{1}H NMR of compound **31** in $CDCl_3$ S30
^{13}C NMR of compound **31** in $CDCl_3$ S30
^{1}H NMR of compound **32** in $CDCl_3$ S31
^{13}C NMR of compound **32** in $CDCl_3$ S31
^{1}H NMR of compound **33** in $CDCl_3$ S32
^{13}C NMR of compound **33** in $CDCl_3$ S32
^{1}H NMR of compound **34** in $CDCl_3$ S33
^{13}C NMR of compound **34** in $CDCl_3$ S33

^{1}H NMR of compound **35** in $CDCl_3$ S34

^{13}C NMR of compound **35** in $CDCl_3$ S34

7. References....................S35

1. General chemistry methods

Dehydroabietic acid was obtained from Pfaltz and Bauer. The other reagents were obtained from Sigma Aldrich Co., VWR International Oy, and Fluorochem. For thin layer chromatography (TLC) Silica gel 60 F254 was used. Flash column chromatography (FCC) was performed with a Biotage High-Performance Flash Chromatography Sp4-system (Uppsala, Sweden) using a 0.1-mm path length flow cell UV detector/recorder module (fixed wavelength: 254 nm), and 10 g, 25 g or 50 g SNAP cartridges (10–50 mL/min flow rate). The ^{1}H NMR and ^{13}C NMR spectra were recorded in either $CDCl_3$, CD_3OD or DMSO-d_6 in a Bruker Ascend 400 spectrometer. The chemical shifts are reported in ppm relative to residual $CHCl_3$ (δ 7.26), CHD2OD (δ 3.31) or DMSO-d_5 (2.50) for ^{1}H NMR. For the ^{13}C NMR spectra, $CDCl_3$ (δ 77.16), CD_3OD (δ 49.00) or DMSO-d_6 (39.52) were used as the internal standards. The coupling constants J are quoted in hertz (Hz). LC-MS analyses were executed with Waters Acquity® UPLC system (Waters, Milford MA, USA) with Acquity PDA detector and Waters Synapt G2 HDMS mass spectrometer (Waters, Milford MA, USA) via an ESI ion source. Samples were analyzed in positive, resolution ion mode. Mass range was set from 100 to 600. Separation was performed in Acquity UPLC® BEH C18 column (1.7 µm, 50 mm × 2.1 mm, Waters, Ireland) in 40 °C. The mobile phase consisted of 0.1% formic acid both in (A) H_2O and (B) acetonitrile (Chromasolv® grade, Sigma-Aldrich, Steinheim, Germany). A linear gradient started at 95% of A and decreased to 10%. Purity of the biologically evaluated compounds was >95%, determined by the UPLC. No unexpected or unusually high safety hazards were encountered.

2. Synthesis

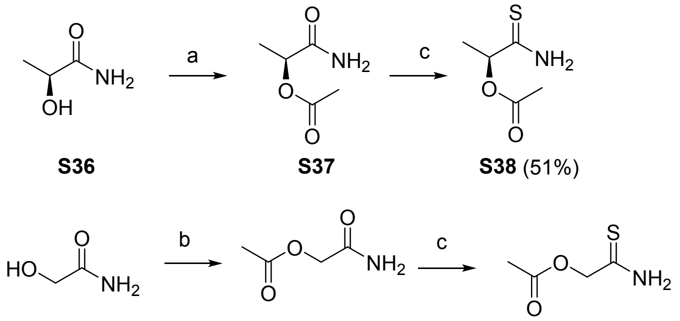

S36 **S37** **S38** (51%)

S39 **S40** **S41** (22%)

Scheme S1. Synthesis of thioamide precursors. Reagents and conditions: (a) Acetyl chloride, 4-methylmorpholine, THF, 0 °C → r.t., 4.5 h. (b) Acetic anhydride, pyridine, 0 °C → r.t., 20 h. (c) Lawesson's reagent, dry 1,4-dioxane, r.t., 1 d. Yields are reported over 2 steps.

(2*S*)-2-(Acetyloxy)propanethioamide (**S38**)

S38 was synthesized according to the literature.[1,2]

^{1}H-NMR ($CDCl_3$, 400 MHz): δ ppm 7.53 (m, 2H), 5.58 (q, J = 6.7 Hz, 1H), 2.15 (s, 3H), 1.63 (d, J = 6.8 Hz, 3H). ^{13}C-NMR ($CDCl_3$, 101 MHz): δ ppm 206.2, 169.0, 76.3, 21.4. HRMS calcd for $C_5H_8NO_2S$. [M-H]$^-$ 146.0276 found 146.0280.

2-(Acetyloxy)ethanethioamide (**S41**)

Glycolamide (0.500 g, 6.66 mmol) was partially dissolved in pyridine (50 mL), and acetic anhydride (3.34 mL, 35.3 mmol) was added dropwise over 15 min on ice bath. The mixture was stirred at 0 °C for 10 min, and at room temperature, for 20 h. The reaction mixture was evaporated to dryness. The crude product was partially dissolved in dry 1,4-dioxane and Lawesson's reagent (1.35 g, 3.33 mmol) was added. The resulting mixture was stirred at room temperature, under argon, for 16 h. The solvent was evaporated and water (30 mL) was added to the residue on ice bath. The mixture was extracted with diethyl ether (3 × 25 mL), and the organic phases were combined, washed with saturated aqueous $NaHCO_3$ solution (30 mL) and brine (15 mL). The organic phase was dried with anhydrous Na_2SO_4, filtered, and concentrated *in vacuo* to give a yellow oil. The crude was purified with automated column chromatography eluting *n*-heptane/ethyl acetate gradient (12 → 100% ethyl acetate) to give **S41** as a white solid (196 mg, 22%).

^{1}H-NMR (DMSO-d_6, 400 MHz): δ ppm 9.90 (brs, 1H), 9.23 (brs, 1H), 4.70 (s, 2H), 2.10 (s, 3H). ^{13}C-NMR (DMSO-d_6, 101 MHz): δ ppm 199.7, 169.7, 67.7, 20.7. HRMS calcd for $C_4H_6NO_2S$. [M-H]$^-$ 132.0119 found 132.0120.

4-(Abieta-8,11,13-trien-12-yl)thiazole (20)

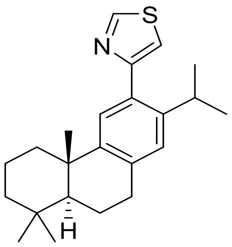

Crude **19** (0.120 g), thioformamide (34.0 mg, 0.555 mmol) and anhydrous 1,4-dioxane (3.5 mL) were irradiated under microwaves, at 100 °C, for 10 min. The reaction mixture was diluted with ethyl acetate (30 mL) and washed with a 1 M solution of NaOH in H_2O (30 mL). The aqueous phase was extracted with ethyl acetate (2 × 15 mL) and the organic phases were combined, washed with brine (15 mL), dried with anhydrous Na_2SO_4, filtered and concentrated *in vacuo* to give a brown solid. The crude was purified with automated column chromatography eluting *n*-heptane/ethyl acetate gradient (1 → 10% ethyl acetate) to give **20** as a colorless solid (36.9 mg, 30% over 2 steps).

^{1}H-NMR ($CDCl_3$, 400 MHz): δ ppm 8.87 (d, J = 2.0 Hz, 1H) 7.27 (s, 1H), 7.20 (d, J = 2.0 Hz, 1H), 7.05 (s, 1H), 3.21 (hept, J = 6.9 Hz, 1H), 2.93 (m, 2H), 2.28 (m, 1H), 1.91 (m, 1H), 1.74 (m, 2H), 1.58 (m, 1H), 1.43 (m, 3H), 1.22 (m, 7H), 1.16 (d, J = 6.9 Hz, 3H), 0.96 (s, 3H), 0.94 (s, 3H). ^{13}C-NMR ($CDCl_3$, 101 MHz): δ ppm 157.5, 151.7, 147.6, 144.0, 135.9, 131.2, 126.5, 126.2, 115.2, 50.5, 41.9, 39.0, 37.7, 33.6, 33.5, 30.5, 29.1, 25.0, 24.3, 24.2, 21.8, 19.4, 19.2. HRMS calcd for $C_{23}H_{32}NS$. $[M+H]^+$ 354.2255 found 354.2256.

2-Methyl-4-(abieta-8,11,13-trien-12-yl)thiazole (21)

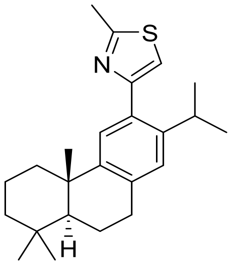

Crude **19** (0.120 g), thioacetamide (42.0 mg, 0.5547 mmol) and EtOH (3.5 mL) were irradiated under microwaves, at 120 °C, for 30 min. The reaction mixture was diluted with ethyl acetate (30 mL) and washed with a 1 M solution of NaOH in H_2O (30 mL). The aqueous phase was extracted with ethyl acetate (2 × 15 mL) and the organic phases were combined, washed with brine (25 mL), dried with anhydrous Na_2SO_4, filtered and concentrated *in vacuo* to give a brown oil. The crude was purified with automated column chromatography

eluting *n*-heptane/ethyl acetate gradient (1 → 10% ethyl acetate) to give **21** as a colorless oil (30.6 mg, 30% over 2 steps).

^{1}H-NMR ($CDCl_3$, 400 MHz): δppm 7.23 (s, 1H), 7.02 (s, 1H), 6.95 (s, 1H), 3.19 (hept, J = 6.9 Hz, 1H), 2.91 (m, 2H), 2.77 (s, 3H), 2.27 (m, 1H), 1.89 (m, 1H), 1.72 (m, 2H), 1.57 (m, 1H), 1.44 (m, 2H), 1.35 (dd, J_1 = 12.4 Hz, J_2 = 2.4 Hz, 1H), 1.21 (m, 7H), 1.14 (d, J = 6.9 Hz, 3H), 0.95 (s, 3H), 0.93 (s, 3H). ^{13}C-NMR ($CDCl_3$, 101 MHz): δppm 164.6, 156.1, 147.4, 144.0, 135.7, 131.6, 126.3, 126.0, 114.8, 50.5, 41.9, 38.9, 37.6, 33.6, 33.5, 30.4, 29.1, 25.0, 24.4, 24.2, 21.8, 19.4, 19.3. HRMS calcd for $C_{24}H_{34}NS$. $[M+H]^+$ 368.2412 found 368.2413.

4-(Abieta-8,11,13-trien-12-yl)-2-aminothiazole (22)

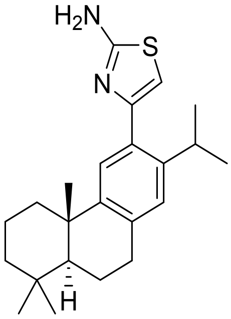

Crude **19** (0.115 g), thiourea (40.0 mg, 0.532 mmol) and EtOH (3.4 mL) were irradiated under microwaves, at 120 °C, for 2 h. The reaction mixture was diluted with ethyl acetate (30 mL) and washed with a 1 M solution of NaOH in H_2O (30 mL). The aqueous phase was extracted with ethyl acetate (2 × 15 mL) and the organic phases were combined, washed with brine (15 mL), dried with anhydrous Na_2SO_4, filtered and concentrated *in vacuo* to give a light brown solid. The crude was purified with automated column chromatography eluting *n*-hexane/ethyl acetate gradient (5 → 40% ethyl acetate) to give **22** as a colorless oil (56.5 mg, 58% over 2 steps).

^{1}H-NMR ($CDCl_3$, 400 MHz): δppm 7.22 (s, 1H), 6.99 (s, 1H), 6.32 (s, 1H), 5.15 (brs, 2H), 3.28 (hept, J = 6.9 Hz, 1H), 2.90 (m, 2H), 2.28 (m, 1H), 1.89 (m, 1H), 1.72 (m, 2H), 1.58 (m, 1H) 1.41 (m, 3H), 1.19 (m, 10H), 0.95 (s, 3H), 0.93 (s, 3H). ^{13}C-NMR ($CDCl_3$, 101 MHz): δppm 166.4, 152.2, 147.4, 143.9, 135.5, 131.8, 126.1, 126.0, 105.0, 50.5, 41.9, 39.0, 37.6, 33.6, 33.5, 30.4, 29.0, 25.0, 24.4, 24.2, 21.8, 19.4, 19.2. HRMS calcd for $C_{23}H_{33}N_2S$. $[M+H]^+$ 369.2364 found 369.2363.

Methyl (*S*)-12-[2-(1-acetoxyethyl)thiazol-4-yl]abieta-8,11,13-trien-18-oate (25)

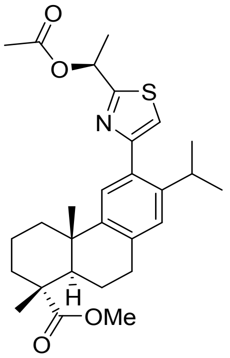

Crude **23** (0.100 mg), **S38** (64.0 mg, 0.433 mmol) and EtOH (2.9 mL) were irradiated under microwaves, at 120 °C, for 30 min. The reaction mixture was diluted with ethyl acetate (25 mL) and washed with a 1 M solution of NaOH in H_2O (25 mL). The aqueous phase was extracted with ethyl acetate (2 × 15 mL) and the organic phases were combined, washed with brine (20 mL), dried with anhydrous Na_2SO_4, filtered and concentrated *in vacuo* to give a brown oil. The crude was purified with automated column chromatography eluting *n*-hexane/ethyl acetate gradient (2 → 20% ethyl acetate) to give **25** as a colorless oil (28.1 mg, 27% over 2 steps).

^{1}H-NMR ($CDCl_3$, 400 MHz): δppm 7.22 (s, 1H), 7.11 (s, 1H), 7.03 (s, 1H), 6.21 (q, J = 6.6 Hz, 1H), 3.67 (s, 3H), 3.15 (hept, J = 6.9 Hz, 1H), 2.92 (dd, J_1 = 8.9, J_2 = 4.6 Hz, 2H), 2.27 (m, 2H), 2.17 (s, 3H), 1.76 (m, 5H), 1.73 (d, J = 6.6 Hz, 3H), 1.49 (m, 2H), 1.28 (s, 3H), 1.23 (s, 3H), 1.20 (d, J = 6.9 Hz, 3H), 1.15 (d, J = 6.9 Hz, 3H). ^{13}C-NMR ($CDCl_3$, 101 MHz): δppm 179.2, 170.1, 168.8, 156.5, 145.8, 144.4, 135.7, 131.4, 126.3, 126.2, 115.6, 70.0, 52.1, 47.8, 45.0, 38.1, 37.1, 36.8, 29.9, 29.3, 25.2, 24.3, 24.1, 21.8, 21.3, 20.9, 18.7, 16.7. HRMS calcd for $C_{28}H_{38}NO_4S$. $[M+H]^+$ 484.2522 found 484.2521.

(*S*)-1-[4-(Abieta-8,11,13-trien-12-yl)thiazol-2-yl]ethyl acetate (27)

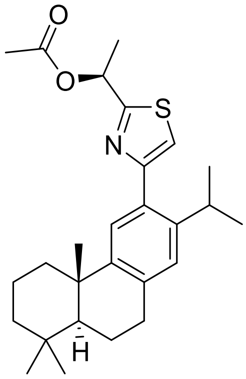

Crude **19** (0.170 g), **S38** (116 mg, 0.786 mmol) and EtOH (5.0 mL) were irradiated under microwaves, at 120 °C, for 30 min. The reaction mixture was diluted with ethyl acetate (40 mL) and washed with a 1 M solution of NaOH in H_2O (40 mL). The aqueous phase was extracted with ethyl acetate (2 × 20 mL) and the organic phases were combined, washed with brine (20 mL), dried with anhydrous Na_2SO_4, filtered and concentrated *in vacuo* to give a brown oil. The crude was purified with automated column chromatography eluting *n*-heptane/ethyl acetate gradient (2 → 20% ethyl acetate) to give **27** as a colorless oil (42.8 mg, 25% over 2 steps).

^{1}H-NMR ($CDCl_3$, 400 MHz): δ ppm 7.23 (s, 1H), 7.11 (s, 1H), 7.04 (s, 1H), 6.21 (q, J = 6.6 Hz, 1H), 3.16 (hept, J = 6.9 Hz, 1H), 2.92 (m, 2H), 2.27 (m, 1H), 2.17 (s, 3H), 1.90 (m, 1H), 1.74 (d, J = 6.6 Hz, 3H), 1.73 (m, 2H), 1.59 (m, 1H), 1.45 (m, 2H), 1.35 (dd, J_1 = 12.4, J_2 = 2.4 Hz, 1H), 1.22 (m, 7H), 1.15 (d, J = 6.9 Hz, 3H), 0.96 (s, 3H), 0.94 (s, 3H). ^{13}C-NMR ($CDCl_3$, 101 MHz): δ ppm 170.1, 168.6, 156.8, 147.5, 144.1, 135.9, 131.3, 126.3, 126.2, 115.5, 70.1, 50.5, 41.9, 38.9, 37.6, 33.6, 33.5, 30.4, 29.3, 25.0, 24.3, 24.1, 21.8, 21.3, 20.9, 19.4, 19.2. HRMS calcd for $C_{27}H_{38}NO_2S$. $[M+H]^+$ 440.2621 found 440.2623.

Methyl (*S*)-12-[2-(1-hydroxyethyl)thiazol-4-yl]abieta-8,11,13-trien-18-oate (28)

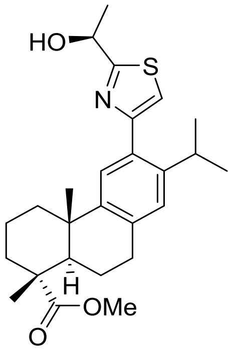

Compound **25** (0.144 g, 0.298 mmol) was dissolved in methanol (2 mL). A 2 M solution of NaOH in H_2O (0.75 mL, 1.5 mmol) was added, and the mixture was stirred, at room temperature, for 2 h. The reaction mixture was diluted with dichloromethane (50 mL) and water (20 mL). The pH of the aqueous phase was adjusted to 8 with a 2 M solution of HCl in H_2O, and the phases were separated. The organic phase was washed with brine (20 mL), dried with anhydrous Na_2SO_4, filtered and concentrated *in vacuo* to give a light brown oil. The crude was purified with automated column chromatography eluting *n*-heptane/ethyl acetate gradient (5 → 40% ethyl acetate) to give **28** as an amorphous colorless solid (84.7 mg, 65%).

^{1}H-NMR ($CDCl_3$, 400 MHz): δ ppm 7.23 (s, 1H), 7.10 (s, 1H), 7.03 (s, 1H), 5.17 (q, J = 6.5 Hz, 1H), 3.67 (s, 3H), 3.15 (hept, J = 6.9 Hz, 1H), 2.92 (dd, J_1 = 8.9, J_2 = 4.5 Hz, 2H), 2.27 (m, 2H), 1.77 (m, 5H), 1.68 (d, J = 6.5 Hz, 3H), 1.54 (m, 1H), 1.44 (m, 1H), 1.28 (s, 3H), 1.23 (s, 3H), 1.19 (d, J = 6.9 Hz, 3H), 1.15 (d, J = 6.9

Hz, 3H), 1H not observed (exchangeable). ^{13}C-NMR ($CDCl_3$, 101 MHz): δ ppm 179.2, 174.0, 156.0, 146.9, 144.4, 135.7, 131.4, 126.3, 126.2, 115.4, 68.2, 52.1, 47.8, 45.0, 38.1, 37.1, 36.8, 29.9, 29.3, 25.2, 24.4, 24.2, 24.1, 21.8, 18.7, 16.7. HRMS calcd for $C_{26}H_{36}NO_3S$. $[M+H]^+$ 442.2416 found 442.2416.

(*S*)-12-[2-(1-Hydroxyethyl)thiazol-4-yl]abieta-8,11,13-trien-18-amide (**29**)

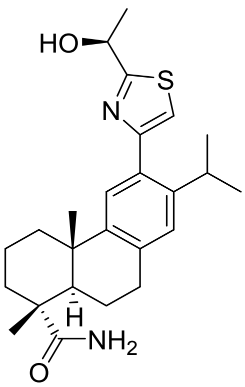

Crude **24** (0.157 g), **S38** (0.125 g, 0.850 mmol) and EtOH (5.0 mL) were irradiated under microwaves, at 120 °C, for 30 min. The reaction mixture was diluted with ethyl acetate (40 mL) and washed with a 1 M solution of NaOH in H_2O (40 mL). The aqueous phase was extracted with ethyl acetate (2 × 20 mL) and the organic phases were combined, washed with brine (20 mL), dried with anhydrous Na_2SO_4, filtered and concentrated *in vacuo* to give a brown oil. The crude was dissolved in methanol (3 mL). A 4 M solution of NaOH in H_2O (0.53 mL) was added, and the mixture was stirred at room temperature for 5 d. The reaction mixture was diluted with dichloromethane (60 mL) and water (30 mL). The pH of the aqueous phase was adjusted to 8 with a 2 M solution of HCl in H_2O, and the phases were separated. The organic phase was washed with brine (20 mL), dried with anhydrous Na_2SO_4, filtered and concentrated *in vacuo* to give a light brown solid. The crude was purified with automated column chromatography eluting *n*-hexane/ethyl acetate gradient (12 → 100% ethyl acetate) to give **29** as an amorphous colorless solid (77.8 mg, 43% over 3 steps).

^1H-NMR ($CDCl_3$, 400 MHz): δ ppm 7.23 (s, 1H), 7.1 (s, 1H), 7.03 (s, 1H), 5.73 (brs, 1H), 5.34 (brs, 1H), 5.19 (q, J = 6.5 Hz), 1H), 3.14 (hept, J = 6.9 Hz, 1H), 2.93 (m, 2H), 2.32 (m, 1H), 2.12 (dd, J_1 = 12.5, J_2 = 2.3 Hz, 1H), 1.70 (m, 7H), 1.69 (d, J = 6.5 Hz, 3H), 1.30 (s, 3H), 1.25 (s, 3H), 1.19 (d, J = 6.9 Hz, 3H), 1.15 (d, J = 6.9 Hz, 3H), 1H not observed (exchangeable). ^{13}C-NMR ($CDCl_3$, 101 MHz): δ ppm 181.2, 174.3, 155.7, 146.9, 144.4, 135.8, 131.1, 126.4, 126.1, 115.5, 68.1, 47.5, 45.6, 38.1, 37.5, 37.2, 29.9, 29.3, 25.2, 24.4, 24.1, 21.2, 18.8, 16.9. HRMS calcd for $C_{25}H_{35}N_2O_2S$. $[M+H]^+$ 427.2419 found 427.2418.

(*S*)-1-[4-(Abieta-8,11,13-trien-12-yl)thiazol-2-yl]ethan-1-ol (30)

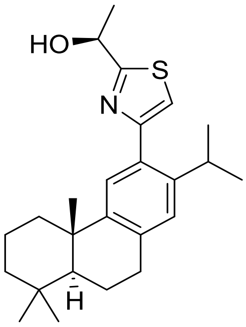

First, intermediate **27** was synthesized like previously described from crude **19** (0.225 g), and it was used without purification. The intermediate was dissolved in methanol (4 mL). A 4 M solution of NaOH in H_2O (0.76 mL) was added, and the mixture was stirred, at room temperature, for 18 h. The reaction mixture was diluted with ethyl acetate (70 mL) and water (30 mL). The pH of the aqueous phase was adjusted to 6 with a 2 M solution of HCl in H_2O and the phases were separated. The organic phase was washed with brine (20 mL), dried with anhydrous Na_2SO_4, filtered and concentrated *in vacuo* to give a brown oil. The crude was purified with automated column chromatography eluting *n*-heptane/ethyl acetate gradient (2 → 20% ethyl acetate) to give **30** as an amorphous colorless solid (109 mg, 45% over 3 steps).

^{1}H-NMR ($CDCl_3$, 400 MHz): δ ppm 7.23 (s, 1H), 7.10 (s, 1H), 7.04 (s, 1H), 5.18 (q, J = 6.5 Hz, 1H), 3.15 (hept, J = 6.9 Hz, 1H), 2.91 (m, 2H), 2.26 (m, 1H), 1.90 (m, 1H), 1.74 (m, 2H), 1.68 (d, J = 6.5 Hz, 3H), 1.58 (m, 1H), 1.45 (m, 2H), 1.35 (dd, J_1 = 12.4, J_2 = 2.4 Hz, 1H), 1.21 (m, 7H), 1.16 (d, J = 6.9 Hz, 3H), 0.95 (s, 3H), 0.94 (s, 3H), 1H not observed (exchangeable). ^{13}C-NMR ($CDCl_3$, 101 MHz): δ ppm 173.9, 156.1, 147.6, 144.0, 136.0, 131.2, 126.3, 126.3, 115.3, 68.2, 50.5, 41.9, 39.0, 37.7, 33.6, 33.5, 30.4, 29.3, 25.0, 24.4, 24.2, 21.8, 19.4, 19.2. HRMS calcd for $C_{25}H_{36}NOS$. $[M+H]^+$ 398.2518 found 398.2516.

Methyl 12-(2-acetylthiazol-4-yl)abieta-8,11,13-trien-18-oate (31)

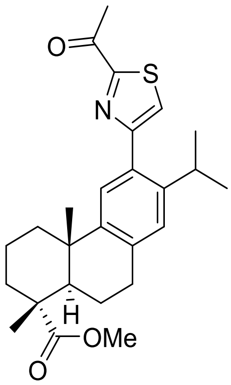

Compound **28** (0.060 g, 0.136 mmol) was dissolved in dichloromethane (0.5 mL) and Dess-Martin periodinane (86.4 mg, 0.204 mmol) was added. The mixture was stirred at room temperature for 4.5 h, after which the reaction mixture was directly purified with automated column chromatography eluting *n*-heptane/ethyl acetate gradient (2 → 20% ethyl acetate) to give **31** as a colorless oil (38 mg, 64%).

^{1}H-NMR ($CDCl_3$, 400 MHz): δ ppm 7.51 (s, 1H), 7.24 (s, 1H), 7.08 (s, 1H), 3.68 (s, 3H), 3.15 (hept, J = 6.9 Hz, 1H), 2.95 (dd, J_1 = 9.0, J_2 = 4.6 Hz, 2H), 2.73 (s, 3H), 2.28 (m, 2H), 1.78 (m, 5H), 1.51 (m, 2H), 1.29 (s, 3H), 1.24 (m, 6H), 1.19 (d, J = 6.9 Hz, 3H). ^{13}C-NMR ($CDCl_3$, 101 MHz): δ ppm 192.2, 179.1, 165.8, 158.9, 147.1, 144.4, 136.3, 130.7, 126.6, 126.1, 123.2, 52.1, 47.7, 44.9, 38.1, 37.1, 36.8, 29.9, 29.4, 26.3, 25.2, 24.3, 24.1, 21.7, 18.6, 16.7. HRMS calcd for $C_{26}H_{34}NO_3S$. $[M+H]^+$ 440.2259 found 440.2258.

12-(2-Acetylthiazol-4-yl)abieta-8,11,13-trien-18-amide (**32**)

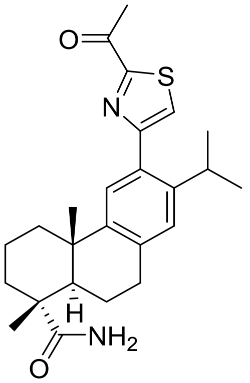

Compound **29** (0.040 g, 0.0938 mmol) was dissolved in dichloromethane (0.7 mL) and Dess-Martin periodinane (59.7 mg, 0.141 mmol) was added. The mixture was stirred, at room temperature, for 26 h, after which the reaction mixture was directly purified with automated column chromatography eluting *n*-hexane/ethyl acetate gradient (12 → 100% ethyl acetate) to give **32** as a colorless oil (25.5 mg, 64%).

^{1}H-NMR ($CDCl_3$, 400 MHz): δ ppm 7.51 (s, 1H), 7.23 (s, 1H), 7.07 (s, 1H), 5.77 (brs, 1H), 5.48 (brs, 1H), 3.14 (hept, J = 6.8 Hz, 1H), 2.95 (m, 2H), 2.74 (s, 3H), 2.31 (m, 1H), 2.16 (dd, J_1 = 12.5, J_2 = 2.3 Hz, 1H), 1.80 (m, 4H), 1.60 (m, 3H), 1.31 (s, 3H), 1.26 (s, 3H), 1.23 (d, J = 6.8 Hz, 3H), 1.18 (d, J = 6.8 Hz, 3H). ^{13}C-NMR ($CDCl_3$, 101 MHz): δ ppm 192.2, 181.3, 165.8, 158.9, 147.1, 144.5, 136.3, 130.7, 126.6, 126.0, 123.2, 47.4, 45.5, 38.1, 37.4, 37.2, 29.9, 29.4, 26.3, 25.3, 24.4, 24.1, 21.2, 18.8, 16.9. HRMS calcd for $C_{25}H_{33}N_2O_2S$. $[M+H]^+$ 425.2263 found 425.2264.

1-[4-(Abieta-8,11,13-trien-12-yl)thiazol-2-yl]ethan-1-one (**33**)

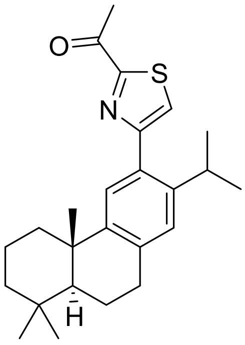

Compound **30** (0.0430 g, 0.108 mmol) was dissolved in dichloromethane (0.5 mL) and Dess-Martin periodinane (69.0 mg, 0.162 mmol) was added. The mixture was stirred at room temperature for 3 h, after which the reaction mixture was directly purified with automated column chromatography eluting *n*-heptane/ethyl acetate gradient (2 → 20% ethyl acetate) to give **33** as a colorless oil (15.3 mg, 37%).

^{1}H-NMR ($CDCl_3$, 400 MHz): δ ppm 7.51 (s, 1H), 7.25 (s, 1H), 7.08 (s, 1H), 3.15 (hept, J = 6.9 Hz, 1H), 2.94 (m, 2H), 2.74 (s, 3H), 2.27 (m, 1H), 1.92 (m, 1H), 1.74 (m, 2H), 1.59 (m, 1H), 1.46 (m, 2H), 1.36 (dd, J_1 = 12.5, J_2 = 2.4 Hz, 1H), 1.25 (m, 4H), 1.22 (s, 3H), 1.19 (d, J = 6.9 Hz, 3H), 0.96 (s, 3H), 0.94 (s, 3H). ^{13}C-NMR ($CDCl_3$, 101 MHz): δ ppm 192.2, 165.7, 159.1, 147.8, 144.1, 136.6, 130.6, 126.5, 126.3, 123.2, 50.5, 41.8, 39.0, 37.7, 33.6, 33.5, 30.4, 29.4, 26.3, 25.1, 24.3, 24.2, 21.8, 19.4, 19.2. HRMS calcd for $C_{25}H_{34}NOS$. $[M+H]^+$ 396.2361 found 396.2361.

Methyl 12-[2-(acetoxymethyl)thiazol-4-yl]abieta-8,11,13-trien-18-oate (**34**)

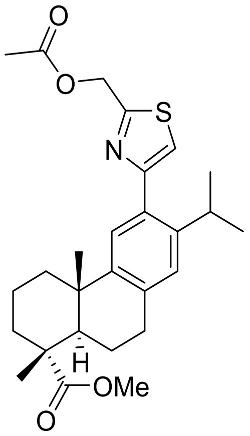

Crude **23** (0.200 g), **S41** (114 mg, 0.859 mmol) and EtOH (5.0 mL) were irradiated under microwaves, at 120 °C, for 30 min. The reaction mixture was diluted with ethyl acetate (50 mL) and washed with a 1 M solution of NaOH in H_2O (50 mL). The aqueous phase was extracted with ethyl acetate (2 × 20 mL) and the organic

phases were combined, washed with brine (20 mL), dried with anhydrous Na_2SO_4, filtered and concentrated *in vacuo* to give a brown oil. The crude was purified with automated column chromatography eluting *n*-heptane/ethyl acetate gradient (3 → 28% ethyl acetate) to give **34** as a colorless oil (54.7 mg, 27% over 2 steps).

^{1}H-NMR ($CDCl_3$, 400 MHz): δ ppm 7.23 (s, 1H), 7.16 (s, 1H), 7.03 (s, 1H), 5.44 (s, 2H), 3.67 (s, 3H), 3.16 (hept, J = 6.9 Hz, 1H), 2.92 (dd, J_1 = 9.0, J_2 = 4.6 Hz, 2H), 2.27 (m, 2H), 2.18 (s, 3H), 1.76 (m, 5H), 1.53 (m, 1H), 1.43 (m, 1H), 1.28 (s, 3H), 1.22 (s, 3H), 1.18 (d, J = 6.9 Hz, 3H), 1.15 (d, J = 6.9 Hz, 3H). ^{13}C-NMR ($CDCl_3$, 101 MHz): δ ppm 179.2, 170.5, 163.4, 156.5, 146.9, 144.3, 135.8, 131.2, 126.3, 126.2, 116.7, 63.0, 52.1, 47.8, 45.0, 38.1, 37.1, 36.8, 29.9, 29.2, 25.2, 24.4, 24.1, 21.8, 21.0, 18.7, 16.7. HRMS calcd for $C_{27}H_{36}NO_4S$. $[M+H]^+$ 470.2365 found 470.2364.

Methyl 12-(2-methylthiazol-4-yl)-7-oxoabieta-8,11,13-trien-18-oate (35)

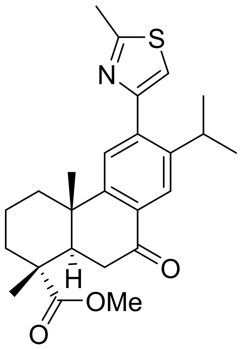

Compound **13** (0.230 g, 0.559 mmol) was suspended in a mixture of acetonitrile (5.3 mL) and water (1.76 mL). *t*-Butyl hydroperoxide (0.38 mL, 2.79 mmol) was added and the mixture was heated to 50 °C. $NaClO_2$ (76.0 mg, 0.671 mmol) was added, and the mixture was heated at 60 °C for 4 days, after which ethyl acetate (2.5 mL) was added and the heating at 60 °C was continued for 3 days. The organic solvents were evaporated to dryness and the residue was diluted with diethyl ether (40 mL) and poured into saturated solution of Na_2SO_3 in H_2O (40 mL), and stirred at room temperature, for 3 h. The phases were separated, and the aqueous phase was extracted with diethyl ether (2 × 20 mL). The organic phases were combined, washed with a saturated solution of $NaHCO_3$ in H_2O (30 mL), water (30 mL) and brine (20 mL). The organic phase was dried with anhydrous Na_2SO_4, filtered and concentrated *in vacuo* to give a colorless solid. The crude product was purified with automated column chromatography eluting *n*-heptane/ethyl acetate gradient (5 → 40% ethyl acetate), after which the product was recrystallized from methanol to give **35** as colorless crystals (12.3 mg, 5.1%).

^{1}H-NMR ($CDCl_3$, 400 MHz): δ ppm 8.04 (s, 1H), 7.39 (s, 1H), 7.08 (s, 1H), 3.65 (s, 3H), 3.26 (hept, J = 6.9 Hz, 1H), 2.79 (s, 3H), 2.72 (m, 2H), 2.36 (m, 2H), 1.74 (m, 5H), 1.35 (s, 3H), 1.28 (s, 3H), 1.23 (d, J = 6.9 Hz,

3H), 1.18 (d, J = 6.9 Hz, 3H). ^{13}C-NMR ($CDCl_3$, 101 MHz): δ ppm 198.4, 178.0, 165.5, 154.5, 152.4, 145.7, 139.8, 130.1, 125.6, 125.1, 116.2, 52.4, 46.9, 43.9, 38.0, 37.4, 37.2, 36.7, 29.4, 24.2, 24.0, 23.8, 19.4, 18.3, 16.6. HRMS calcd for $C_{25}H_{32}NO_3S$. $[M+H]^+$ 426.2107 found 426.2103.

3. Recombinant expression of ABHD16A and ABHD12 in HEK293T cells

cDNA encoding mouse ABHD16A (NM_178592.3), human ABHD16A (NM_021160.3) and human ABHD12 (NM_001042472.3) were synthesized by GenScript and sub-cloned into pEF-BOS-HA.[3] HEK293T cells (ECACC 12022001) were grown in Dulbecco's Modified Eagle's Medium supplemented with 10% foetal bovine serum in a humidified 5% CO_2 atmosphere at 37 °C. Cells were transiently transfected with polyethylenimine (PEI) at a ratio of 3 µl PEI to 1 µg DNA.[4] After 24 hours, cells were scraped into ice-cold PBS (pH 7.4), passed through a 25G needle 20 times and centrifuged at 8000 × g for 10 minutes at 4 °C. Membranes were isolated by passing the supernatant through a 25G needle 20 times, centrifuging at 100,000 × g for 1 h at 4 °C and resuspending the pellet in 500 µL ice-cold PBS.

4. Competitive activity-based protein profiling (cABPP) in HEK293T membrane proteomes

Total membrane proteomes were adjusted to 2 mg/mL in ice-cold PBS (pH 7.4) and incubated with compounds in DMSO at the indicated concentrations for 1 h followed by labelling with 2 µM azido-fluorophosphonate (ActivX™ Azido-FP Serine Hydrolase Probe, Thermo Scientific) for 1 h at room temperature in a total reaction volume of 50 µL. FP-labelled proteins were conjugated to an alkyne IR800 dye (LI-COR, Inc) by end-over-end rotation in an equal volume of click chemistry reaction mix (5 µM IRDye 800CW alkyne infrared dye, 4 mM $CuSO_4$, 400 µM tris[(1-benzyl-1H-1,2,3-triazol-4-yl)methyl]amine, and 8 mM ascorbic acid, in distilled H_2O) for 1 h.[5] Reactions were quenched with 4 X SDS-PAGE loading buffer (200 mM Tris-HCl, pH 6.8, 40 % glycerol, 8 % sodium dodecyl sulphate, 0.4 % Bromophenol Blue, supplemented with 25 mM dithiothreitol) and samples were heated for 5 min at 95 °C, resolved by SDS-PAGE and transferred to nitrocellulose membranes for immunoblotting with a rat anti-HA antibody to detect ABHD16A and ABHD12 (Roche, clone 3F10). Infrared fluorescence was detected using an Odyssey imaging system (LI-COR, Inc). Percentage inhibition was quantified by measuring the difference in the intensity of the Azido-FP band relative to a DMSO control (no compound) and normalized to the HA signal using Image Studio software (LI-COR, Inc). No unexpected or unusually high safety hazards were encountered.

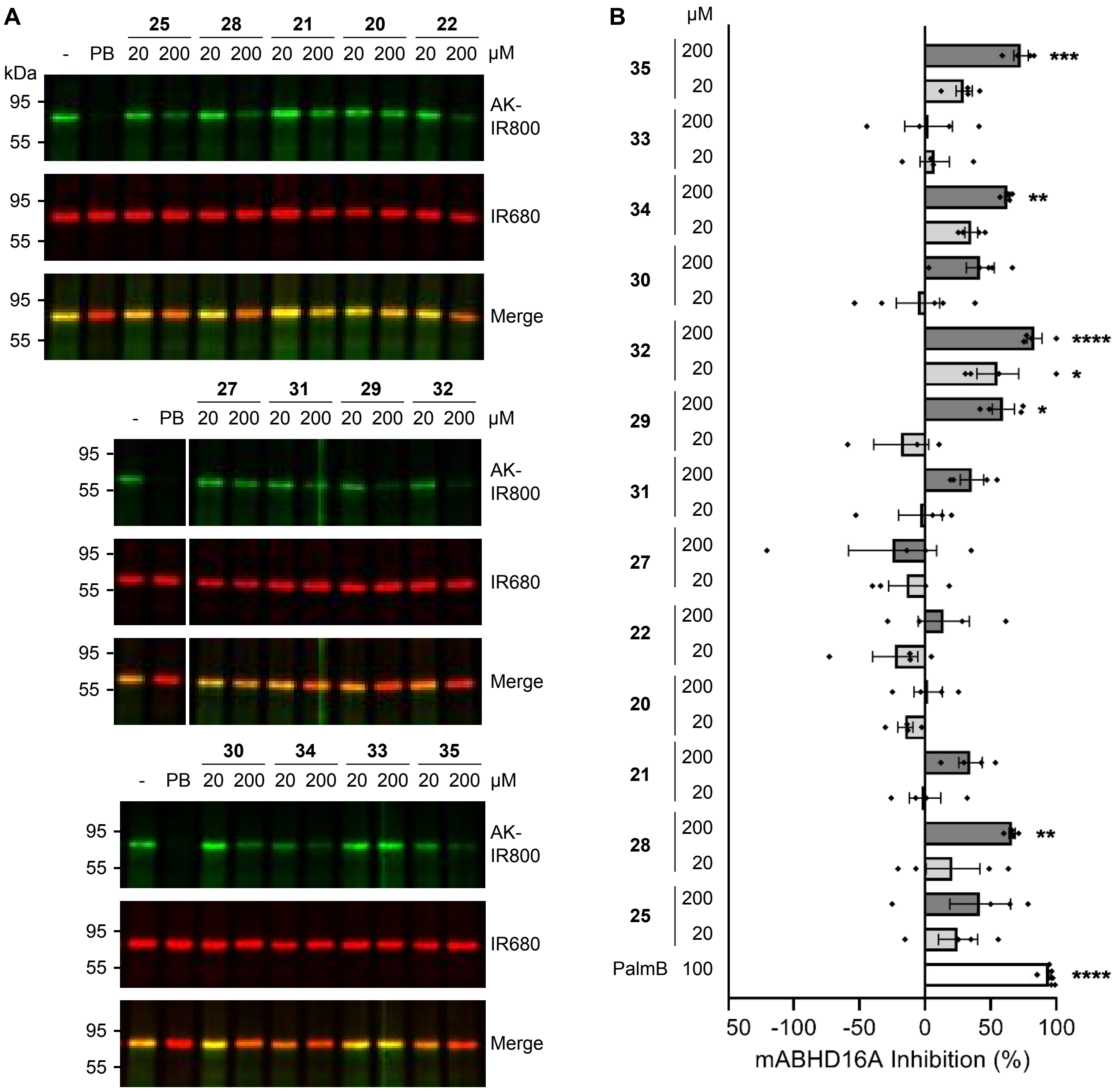

Figure S1. Inhibition of murine ABHD16A by competitive ABPP. HA-tagged mABHD16A enriched from HEK293T total membrane proteomes was incubated with compounds at the indicated concentrations followed by labelling with FP-azide and conjugation by click chemistry to an alkyne-infrared 800 dye (AK-IR800). Anti-HA primary antibody and an anti-rat IR680 secondary antibody were used to detect ABHD16A (IR680). mABHD16A inhibition was calculated by measuring the difference in FP-azide incorporation relative to DMSO control (-), normalized to protein levels. 100 μM Palmostatin B (PB) was used as a positive control. A. Representative immunoblot images are shown: click chemistry signal (Top, AK-IR800), HA (Middle, IR680), and merge (Bottom). The position of molecular weight standards is shown on the left. In the middle panel, the same immunoblots are shown but with one lane removed for clarity. B. Bar chart showing mean percentage mABHD16A inhibition. Individual bullet points represent independent experiments. Error bars represent ± SEM. Statistical significance was determined by one-way ANOVA with Dunnett's post hoc test. For clarity, only statistically significant analysis is shown. *$p<0.05$; **$p<0.01$; ***$p< 0.001$; ****$p<0.0001$.

5. Molecular modeling

To evaluate the binding capability and selectivity of the abietane database from this study towards the human ABHD12 and ABHD16A proteins, we have performed molecular docking calculations using Autodock 4.2.[6] Due to the unavailability of any experimentally determined structure from both these proteins, we have used in this work the available AlphaFold[7] models with reference AF-A0A1A8MD55-F1, and AF-Q8N2K0-F1, respectively corresponding to ABHD16A and ABHD12 proteins. The 3D configurations of all evaluated compounds were generated using the program Balloon[8] to mol2 file format. The compounds and protein models pdbqt file format required to run all molecular docking calculations were generated using respectively, the prepare_ligand4.py and the prepare_receptor4.py scripts from AutoDockTools software package (version 1.5.7).[6] While all compounds were set to be fully flexible, the protein models were set to their fixed configurations. The affinity maps for the atom types found on all dataset compounds were calculated using the program autogrid found in Autodock 4.2 software package. After aligning both structure models based on the catalytic triad positions (composed by a serine, histidine and as aspartate residue), a simulation box was centered on the 3D geometry center space coordinate determined from the positions of these residues. The search space was defined to 66, 70, and 80 points of dimension (with a spacing of 0.375 Angstroms) on the X, Y, and Z axis, to assure that the entire surface of interest was fully covered. In each docking assay, 1000 different poses were generated using Autodock's implemented genetic algorithm. The number of generations was set to 270000, while the size of populations was set to 500. All the remaining parameters in the search mode were set to their default. For each calculation, all generated poses were clustered with a 1.5 Angstrom cut-off. The lowest energy solution of the top-ranking cluster was selected as the most representative docking solution for each compound on each evaluated structural model.

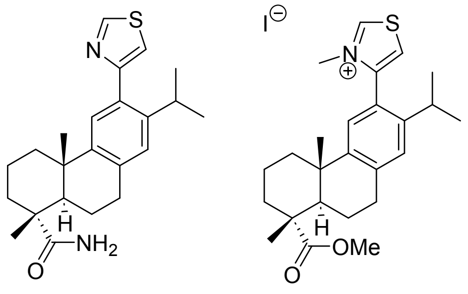

S42 **S43**

Figure S2. Structures of S42 and S43. The preparation of these 12-thiazole abietanes has been reported before.[9]

Table S1. Calculated free energies of binding of the abietane dataset, (-)-tetrahydrolipstatin (**1**), and palmostatin B (**2**), to human ABHD12 and ABHD16A structural models determined with Autodock 4.2.

Compound	ABHD12 binding affinity (kcal mol^{-1})	ABHD16A binding affinity (kcal mol^{-1})
**13**	-9.58	-10.07
**20**	-9.18	-9.73
**21**	-9.59	-10.16
**22**	-9.13	-9.41
**25**	-10.04	-9.71
**27**	-9.84	-10.28
**28**	-9.96	-9.9
**29**	-9.73	-9.79
**30**	-9.61	-9.85
**31**	-9.96	-10.21
**32**	-10.15	-9.83
**33**	-9.71	-10.46
**34**	-9.77	-10.15
**35**	-9.98	-10.06
**S42**	-9.5	-9.27
**S43**	-9.69	-9.91
**1**	-8.71	-8.26
**2**	-7.69	-7.81

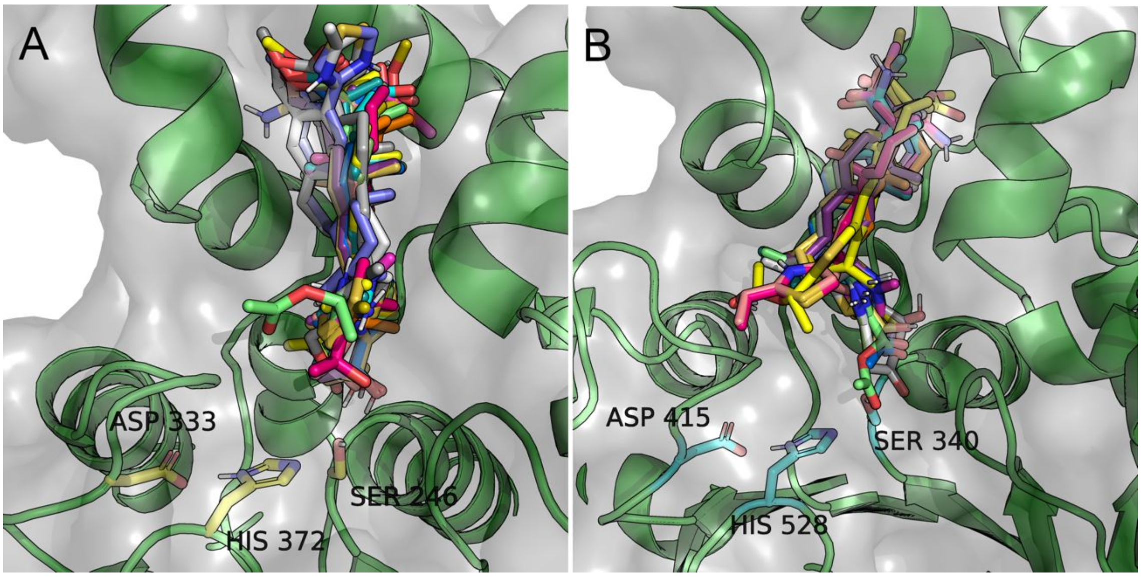

Figure S3. ABHD12 (A) and ABHD16A (B) protein model representations with the catalytic triad highlighted in yellow and cyan sticks respectively, and all the dataset compounds docking solutions represented as coloured sticks.

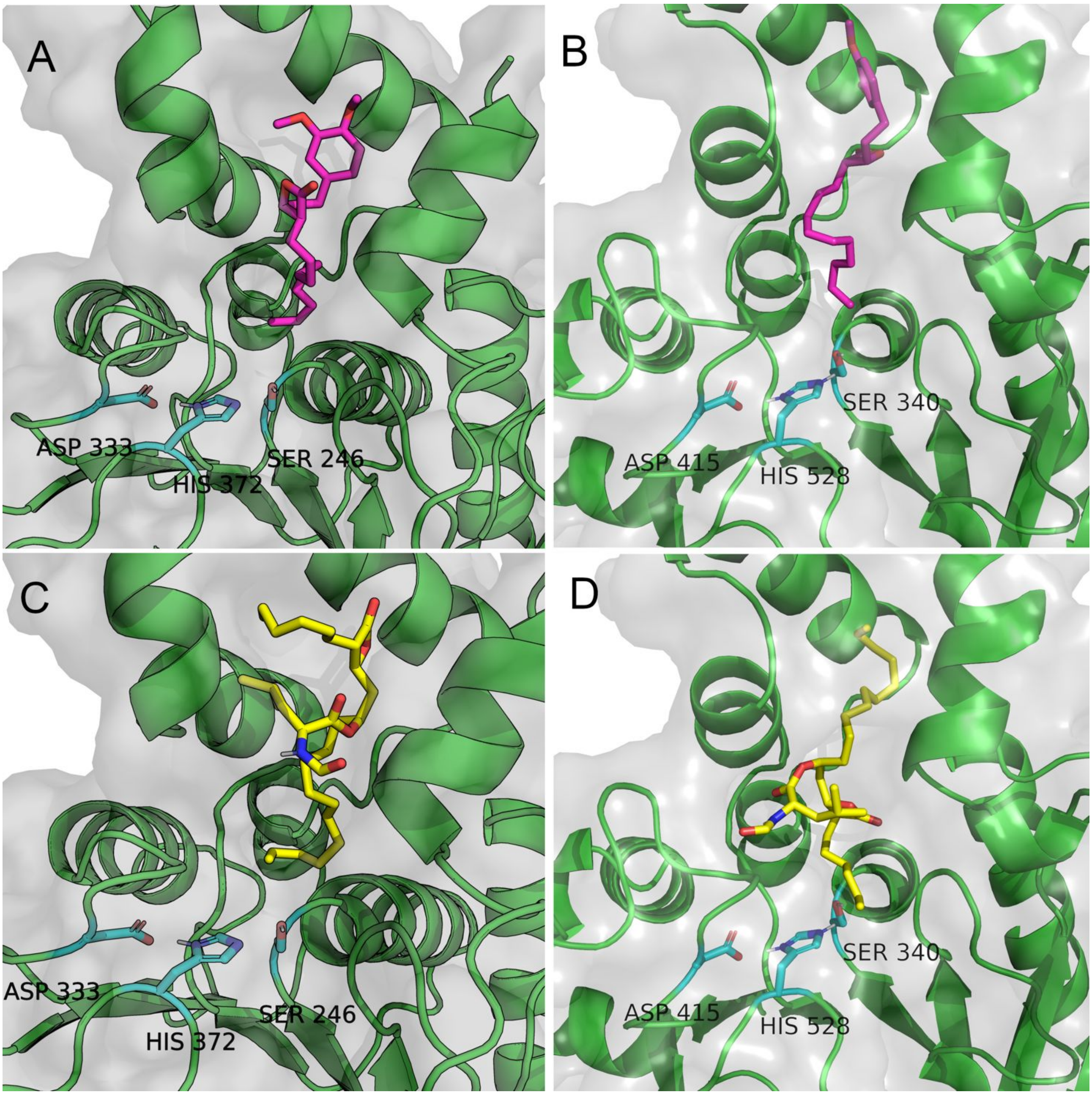

Figure S4. ABHD12 (A,C) and ABHD16A (B,D) protein model representations with the catalytic triad highlighted in cyan sticks, and (-)-tetrahydrolipstatin (**1**) and palmostatin B (**2**), represented in yellow and magenta sticks.

6. NMR spectra

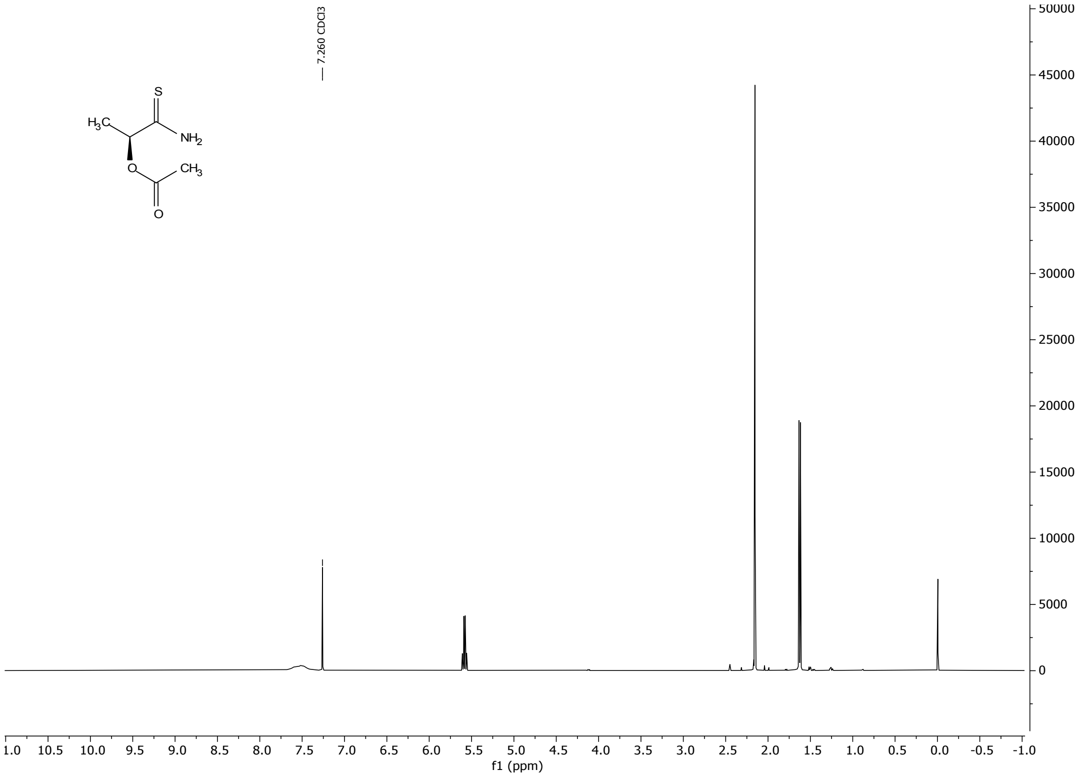

^{1}H NMR of compound **S38** in $CDCl_3$

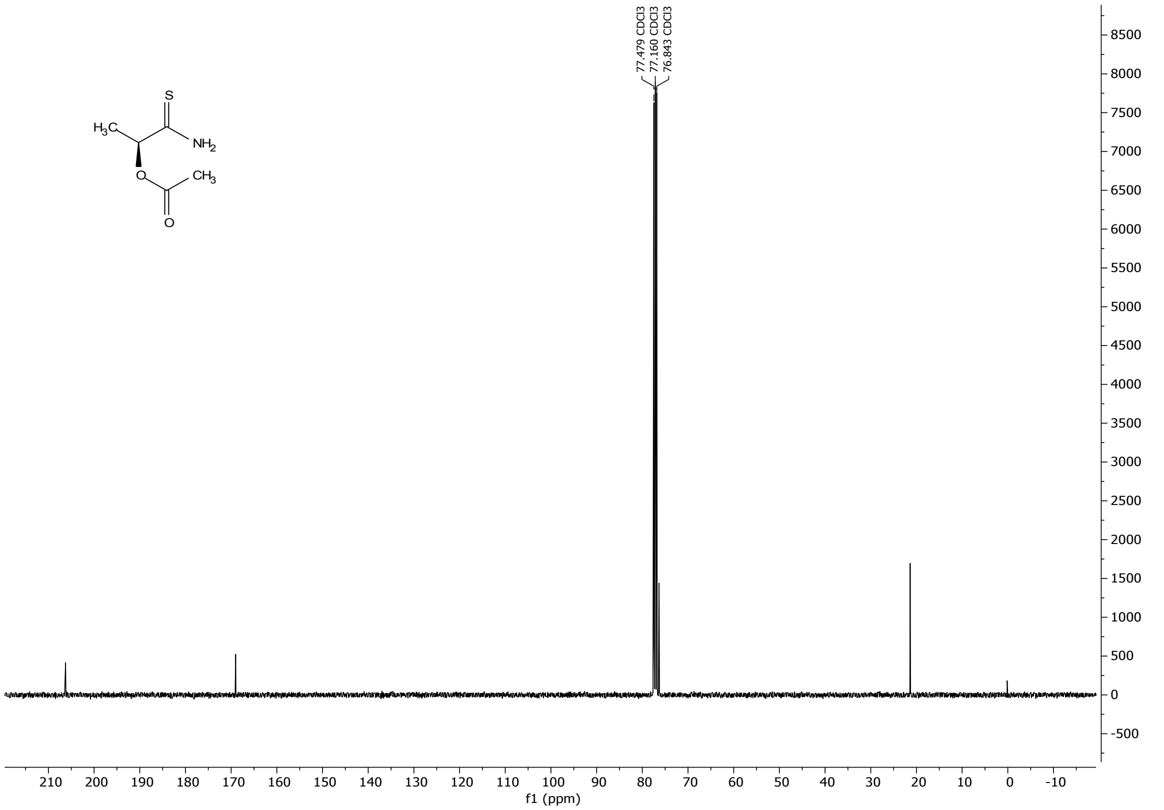

^{13}C NMR of compound **S38** in $CDCl_3$

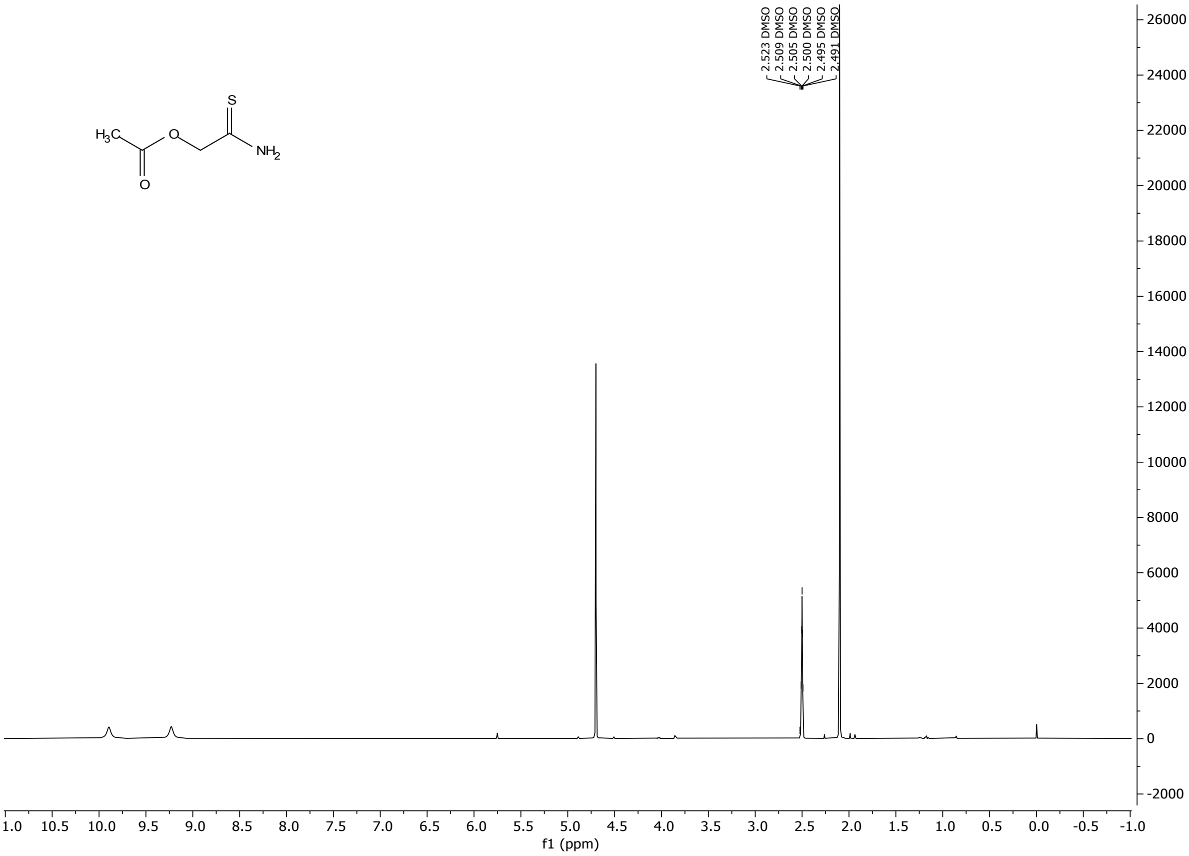

^{1}H NMR of compound **S41** in DMSO-d_6

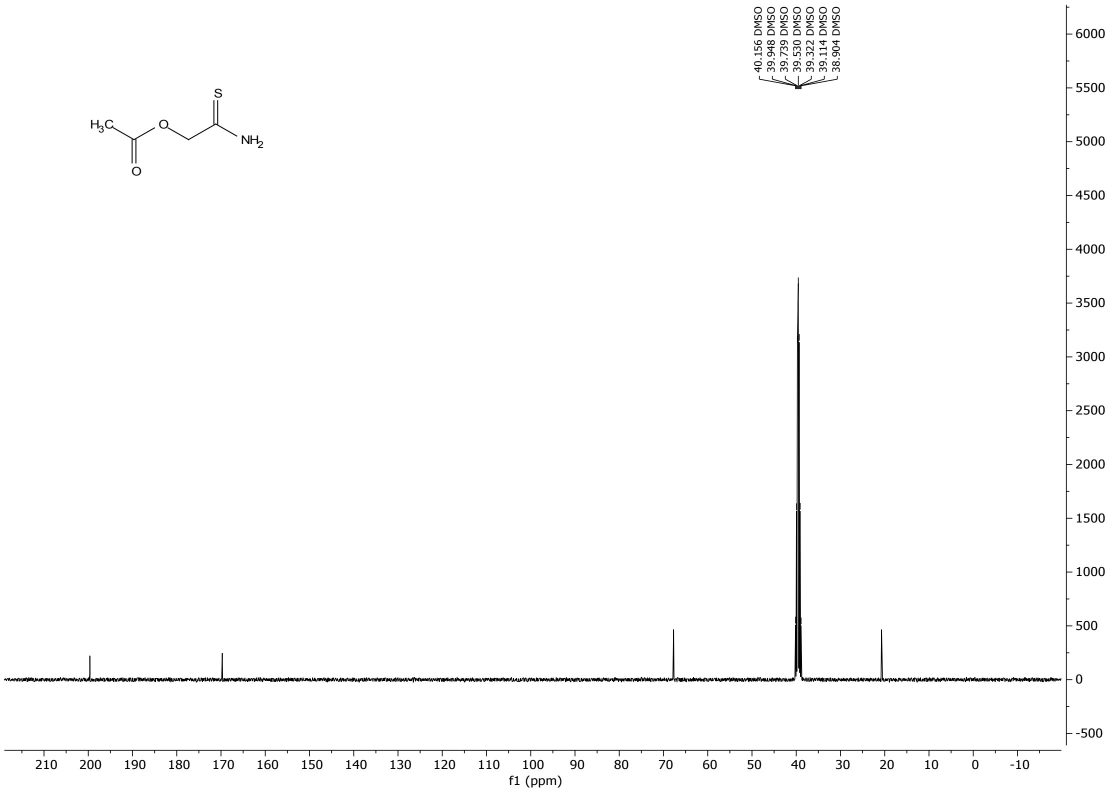

^{13}C NMR of compound **S41** in DMSO-d_6

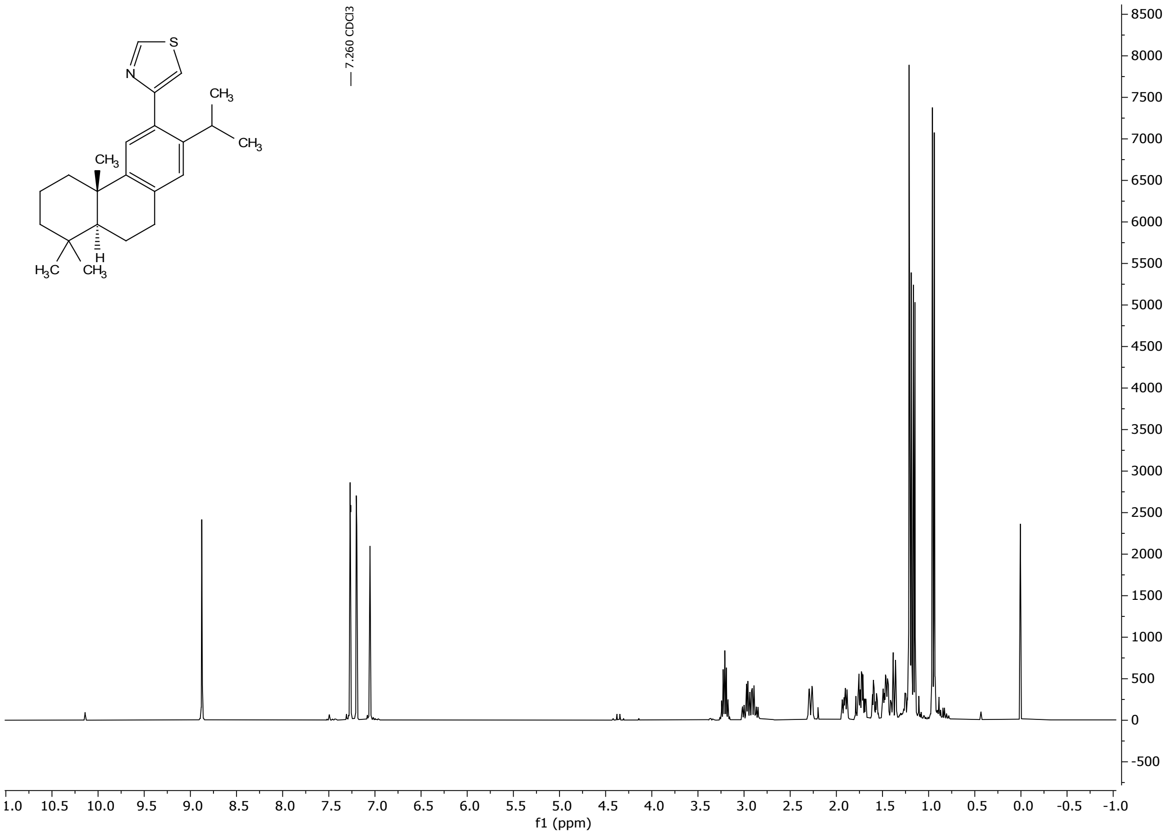

^{1}H NMR of compound **20** in $CDCl_3$

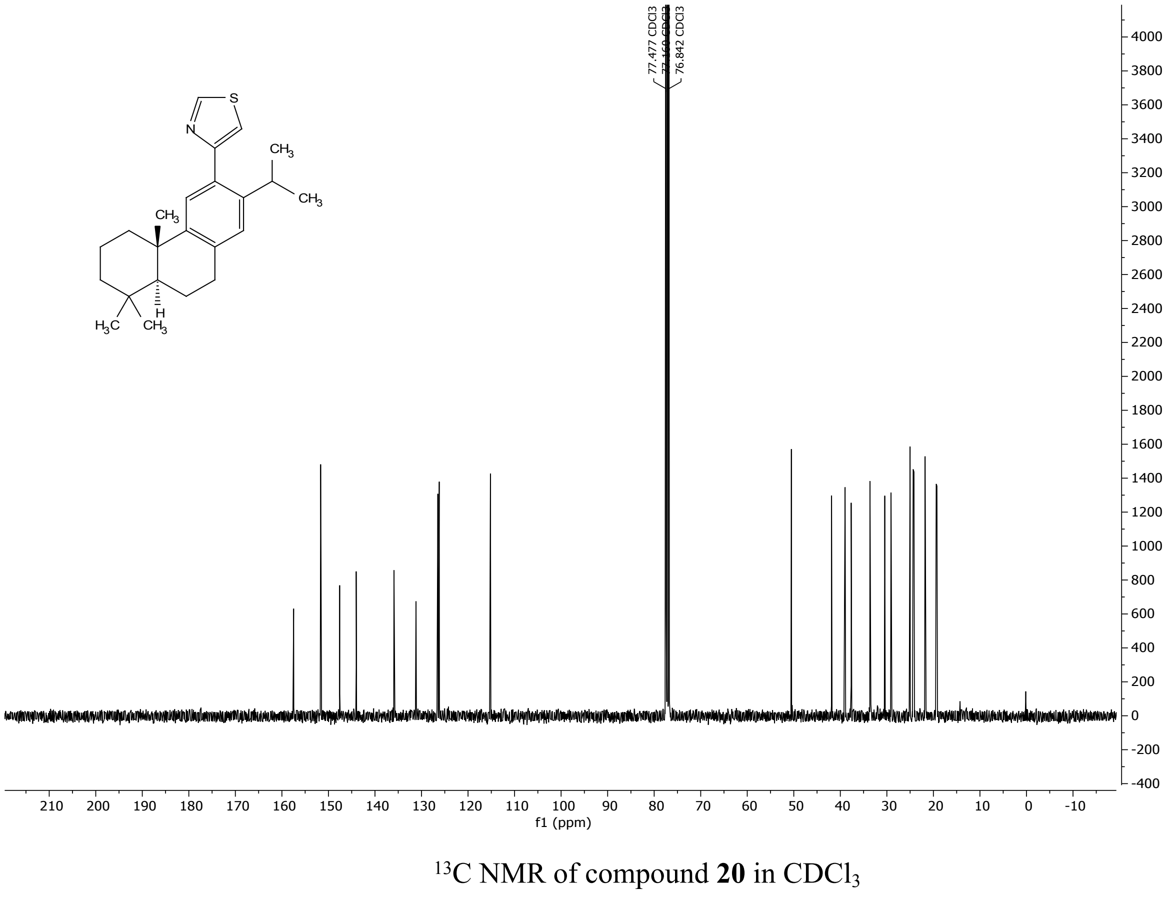

^{13}C NMR of compound **20** in $CDCl_3$

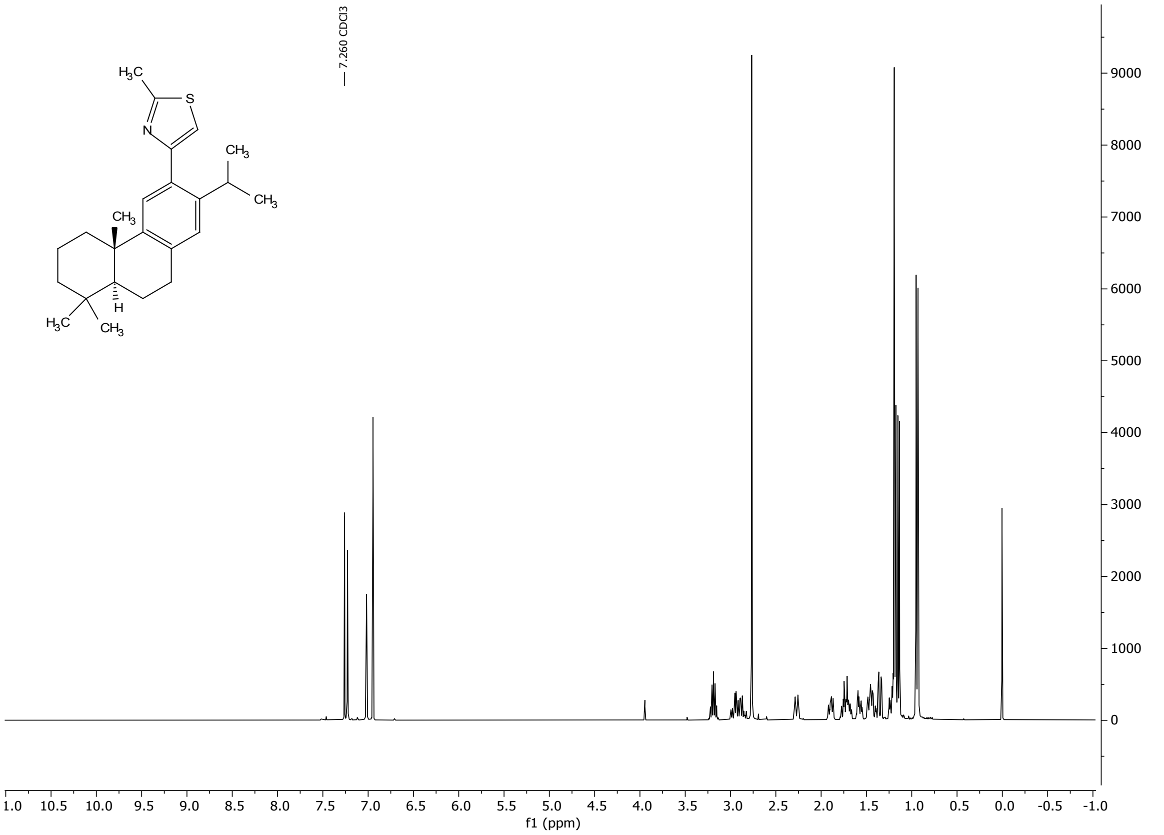

^{1}H NMR of compound **21** in $CDCl_3$

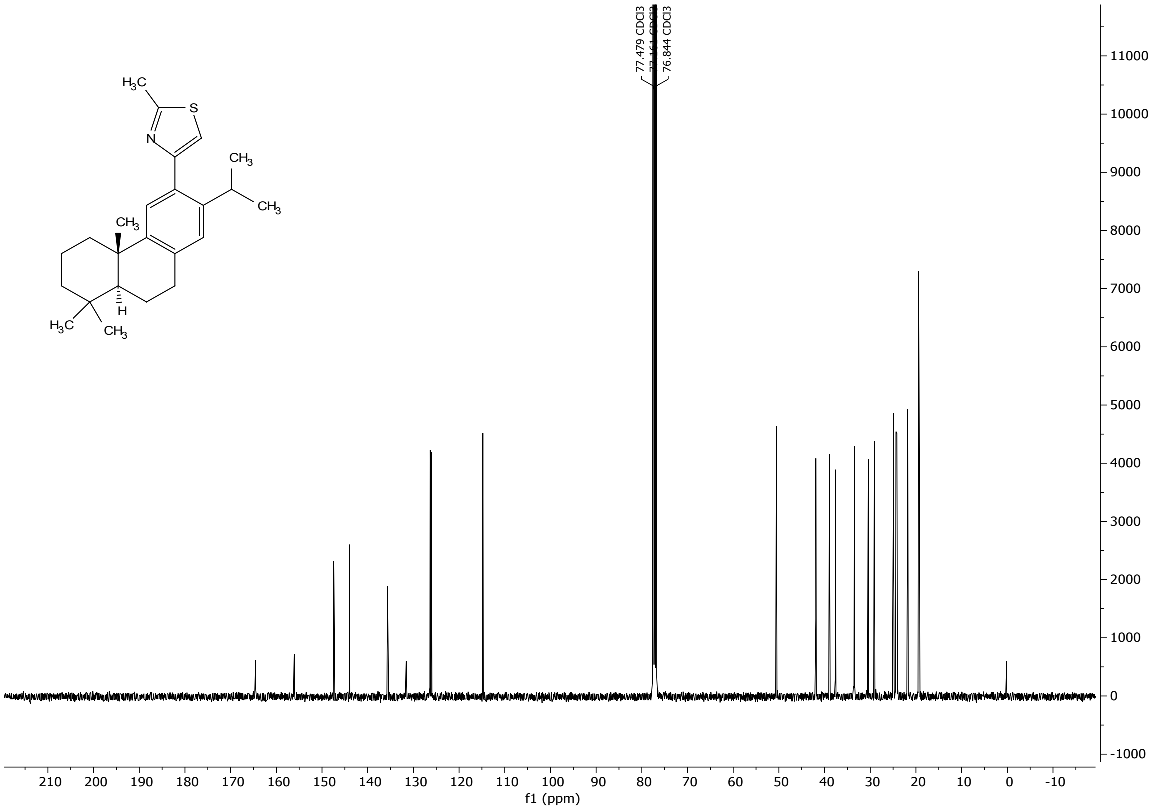

^{13}C NMR of compound **21** in $CDCl_3$

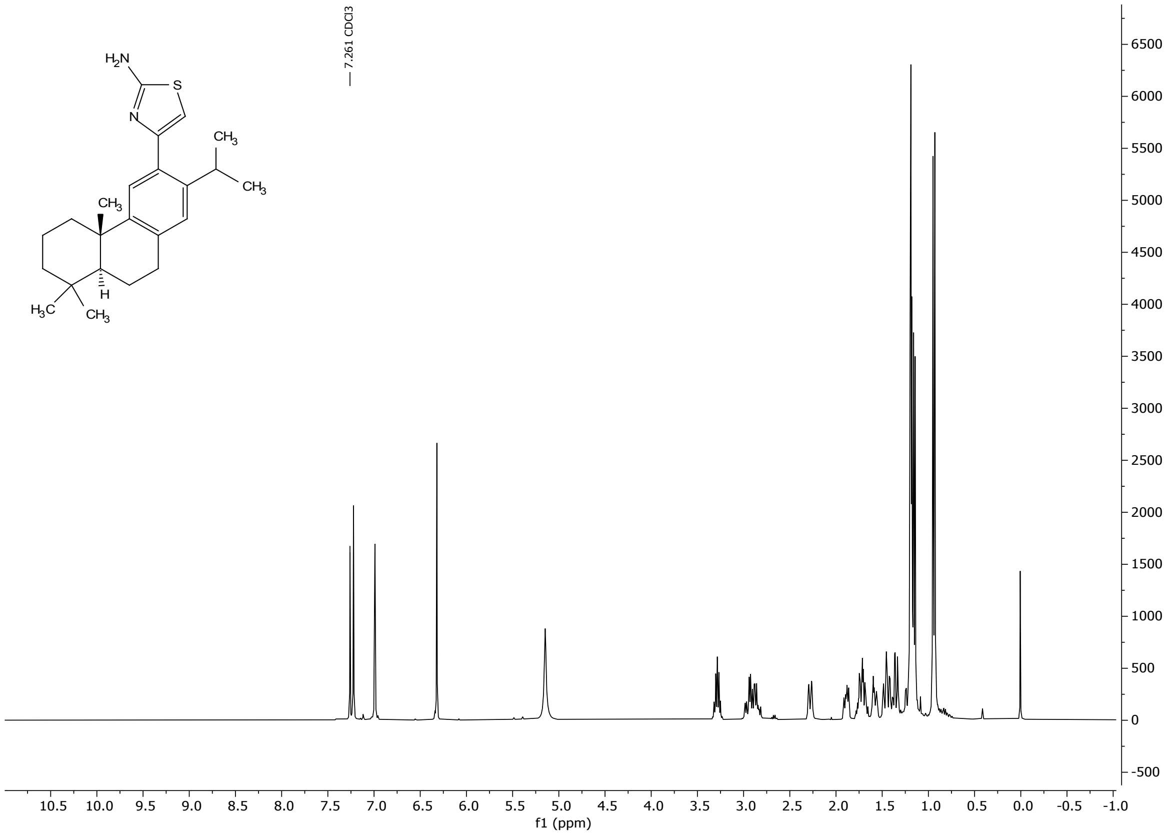

^{1}H NMR of compound **22** in $CDCl_3$

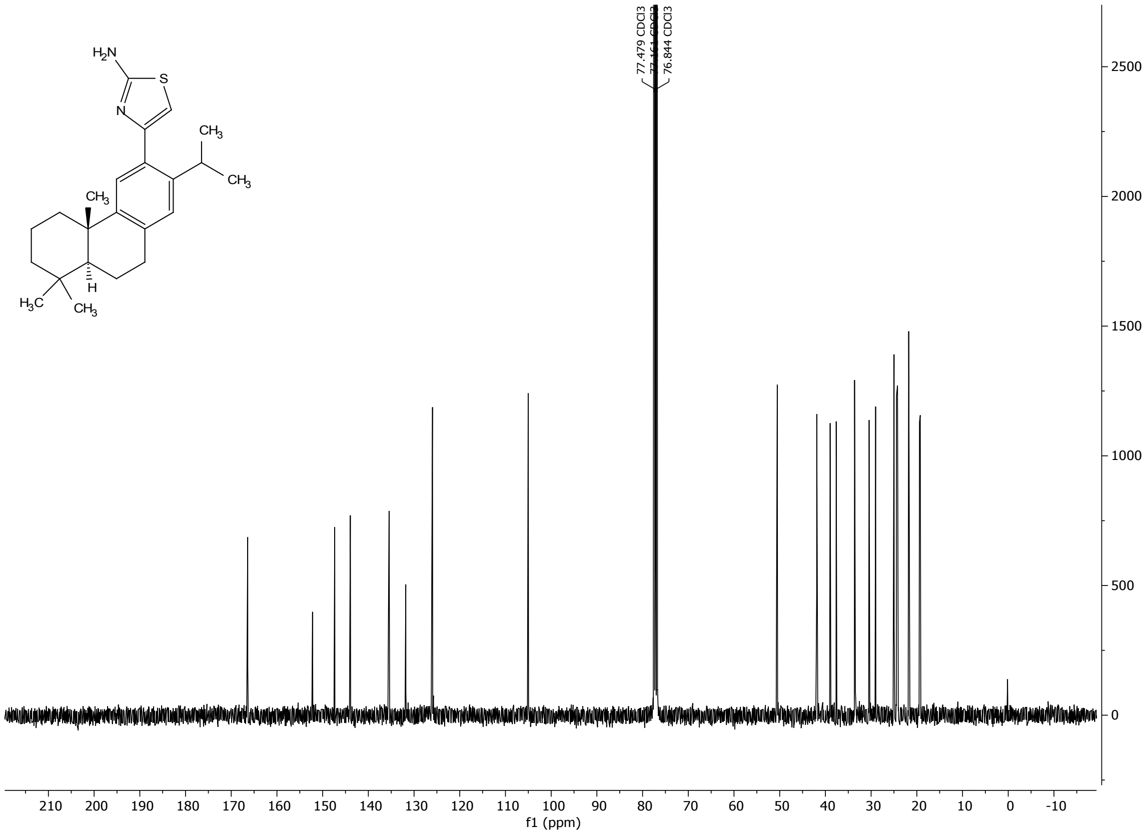

^{13}C NMR of compound **22** in $CDCl_3$

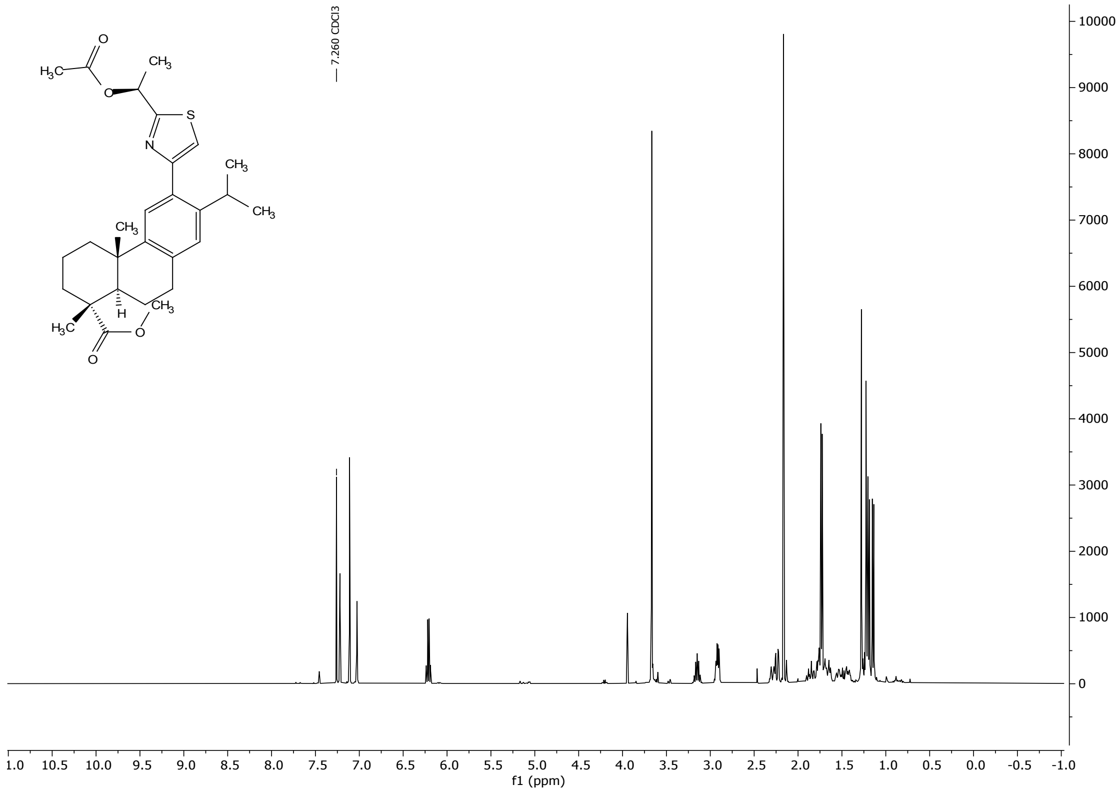

^{1}H NMR of compound **25** in $CDCl_3$

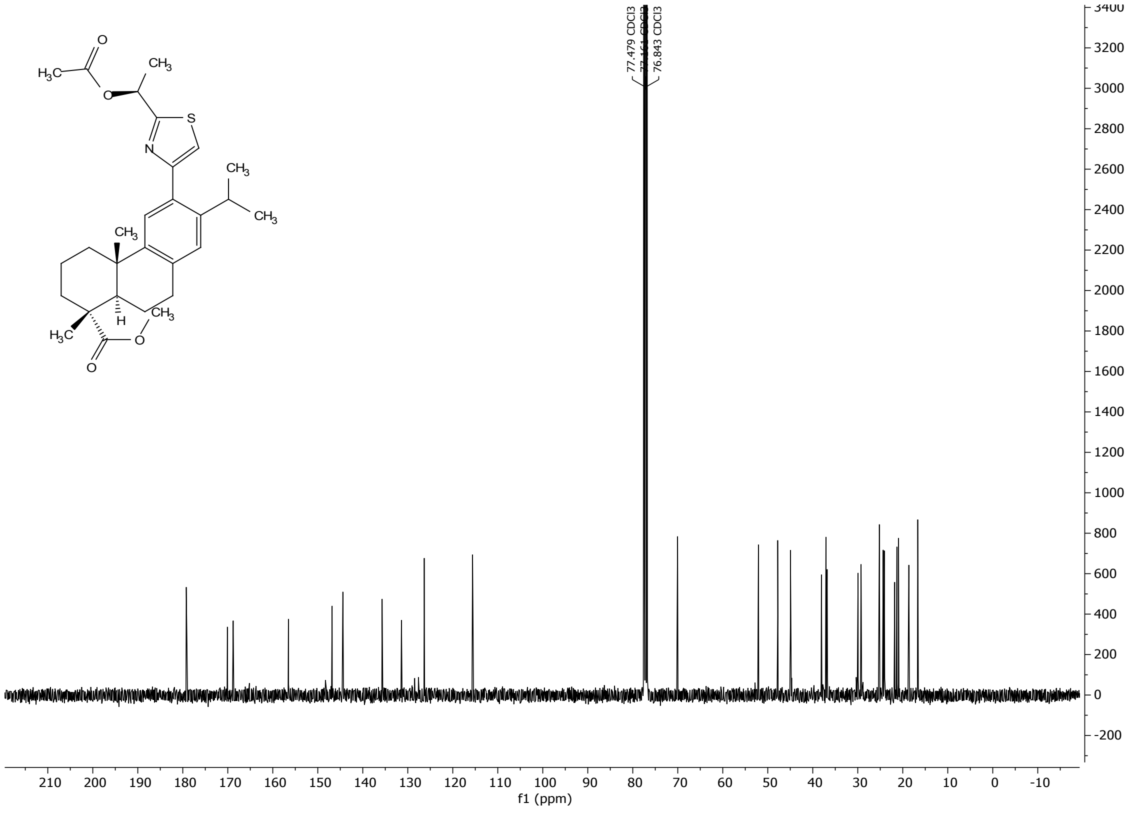

^{13}C NMR of compound **25** in $CDCl_3$

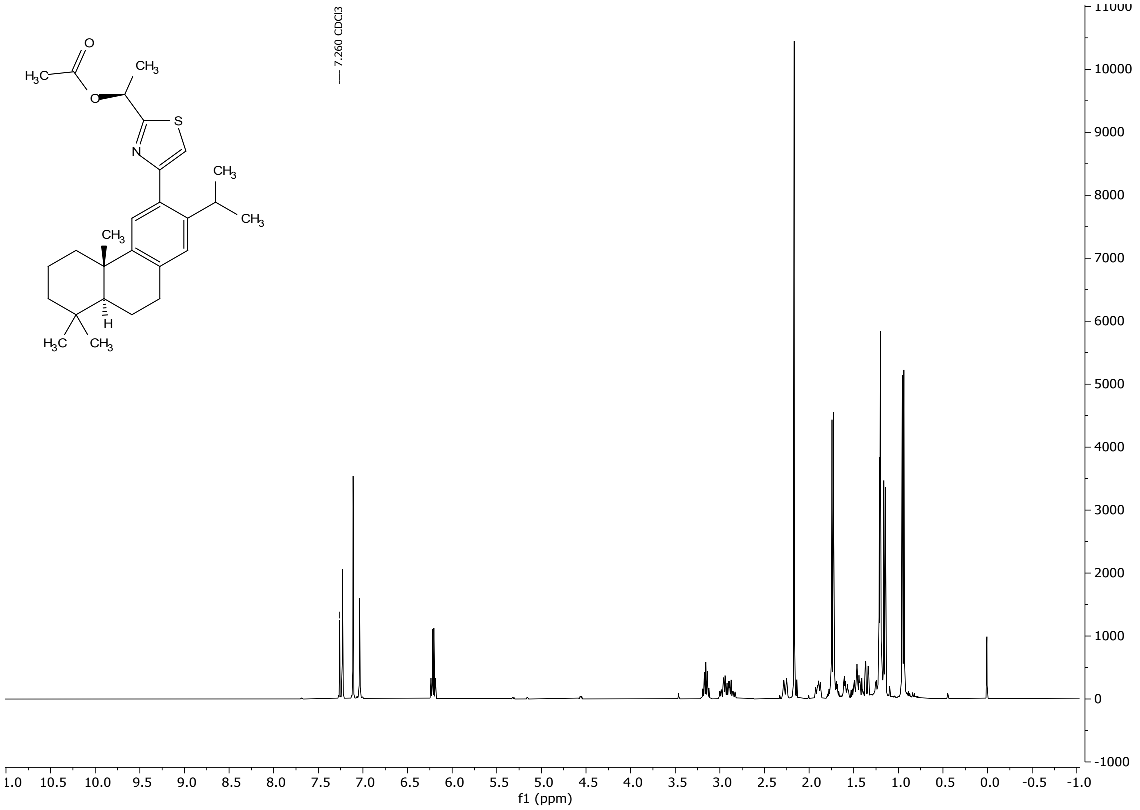

^{1}H NMR of compound **27** in $CDCl_3$

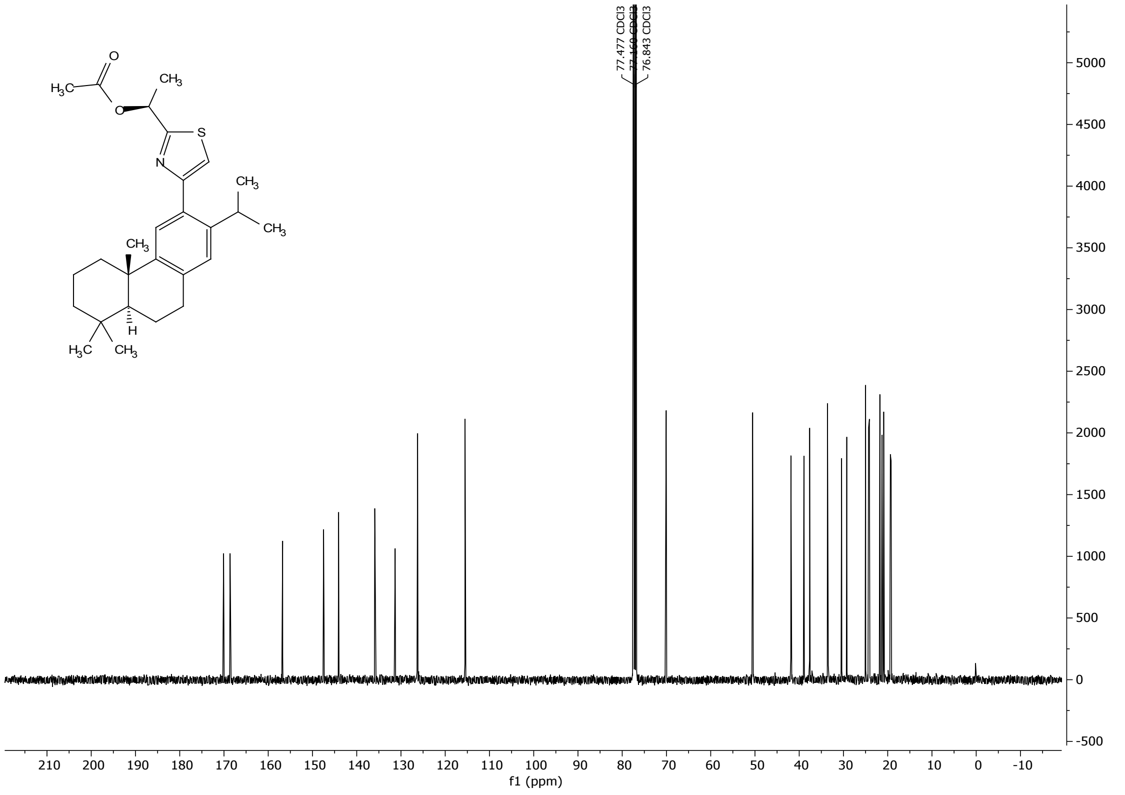

^{13}C NMR of compound **27** in $CDCl_3$

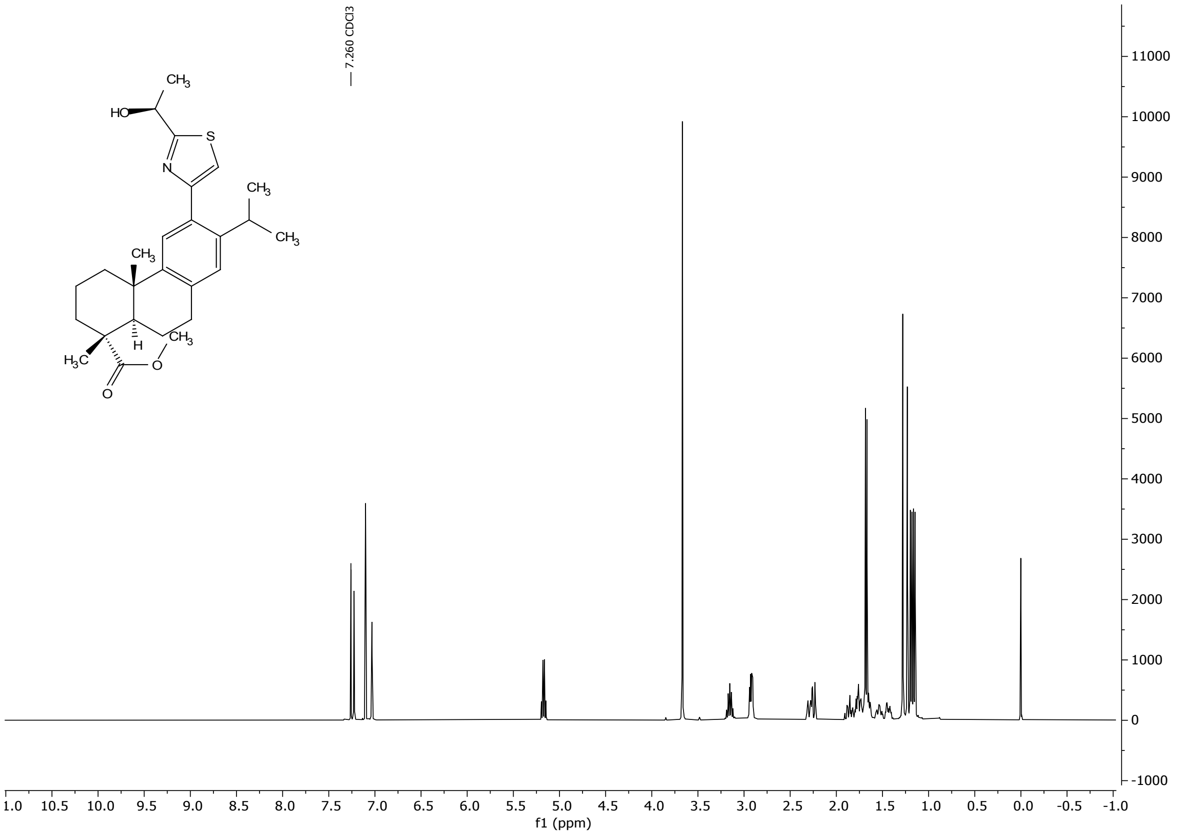

^{1}H NMR of compound **28** in $CDCl_3$

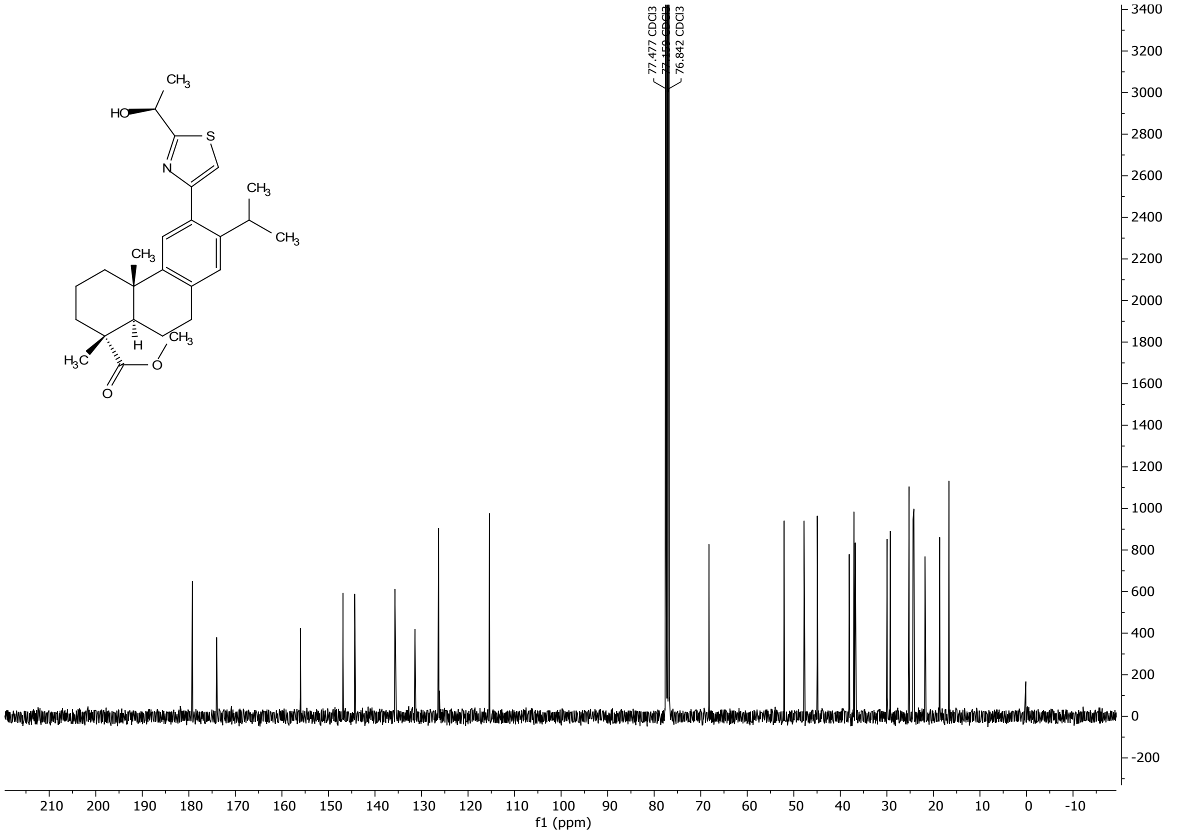

^{13}C NMR of compound **28** in $CDCl_3$

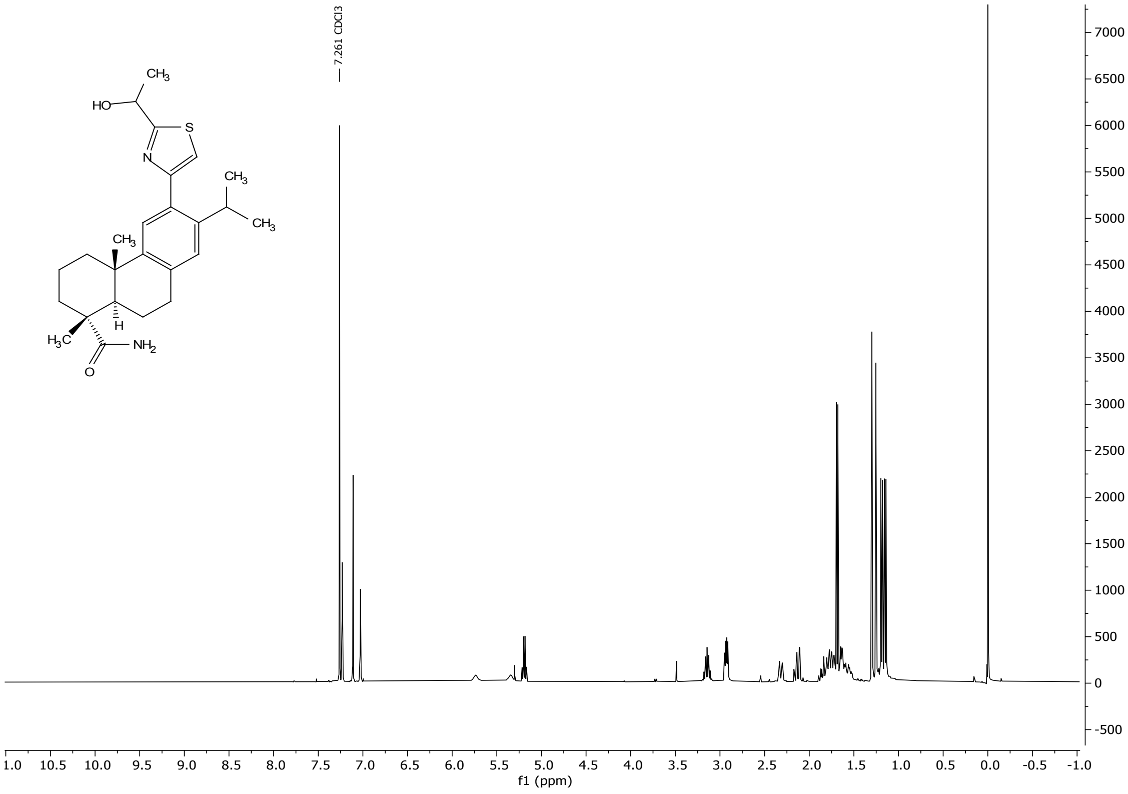

^{1}H NMR of compound **29** in $CDCl_3$

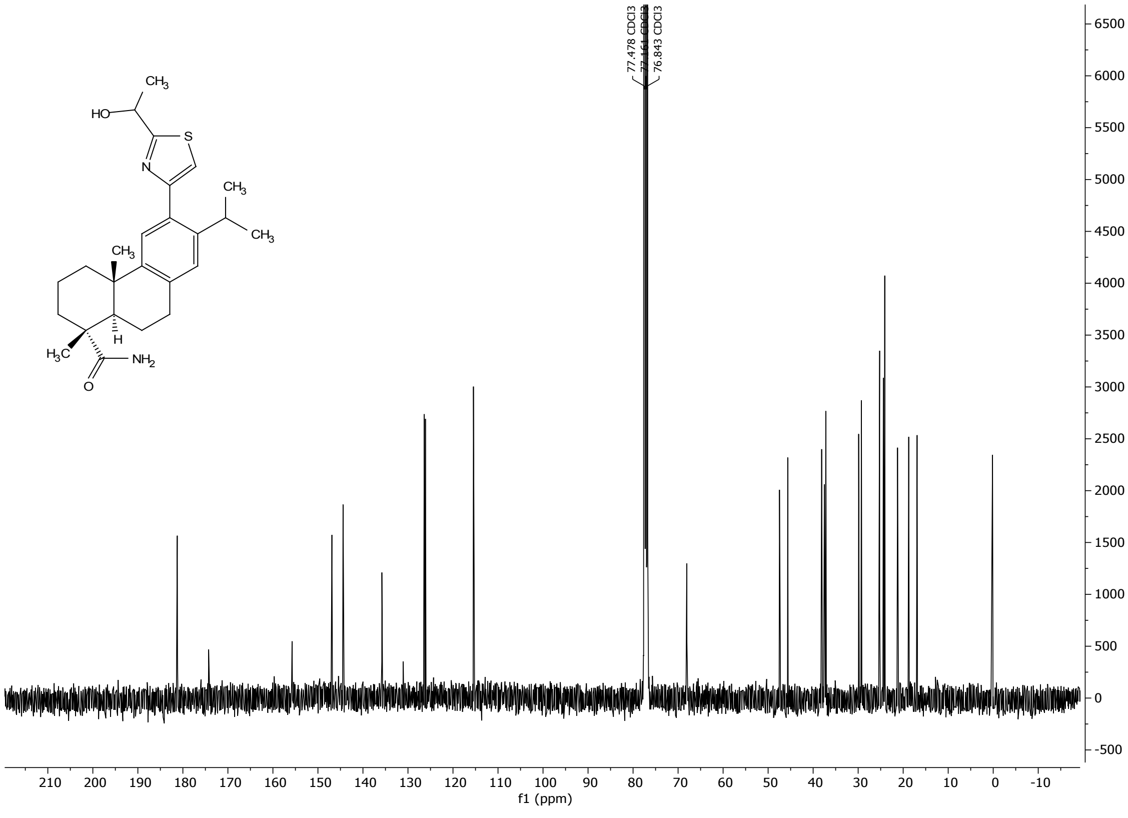

^{13}C NMR of compound **29** in $CDCl_3$

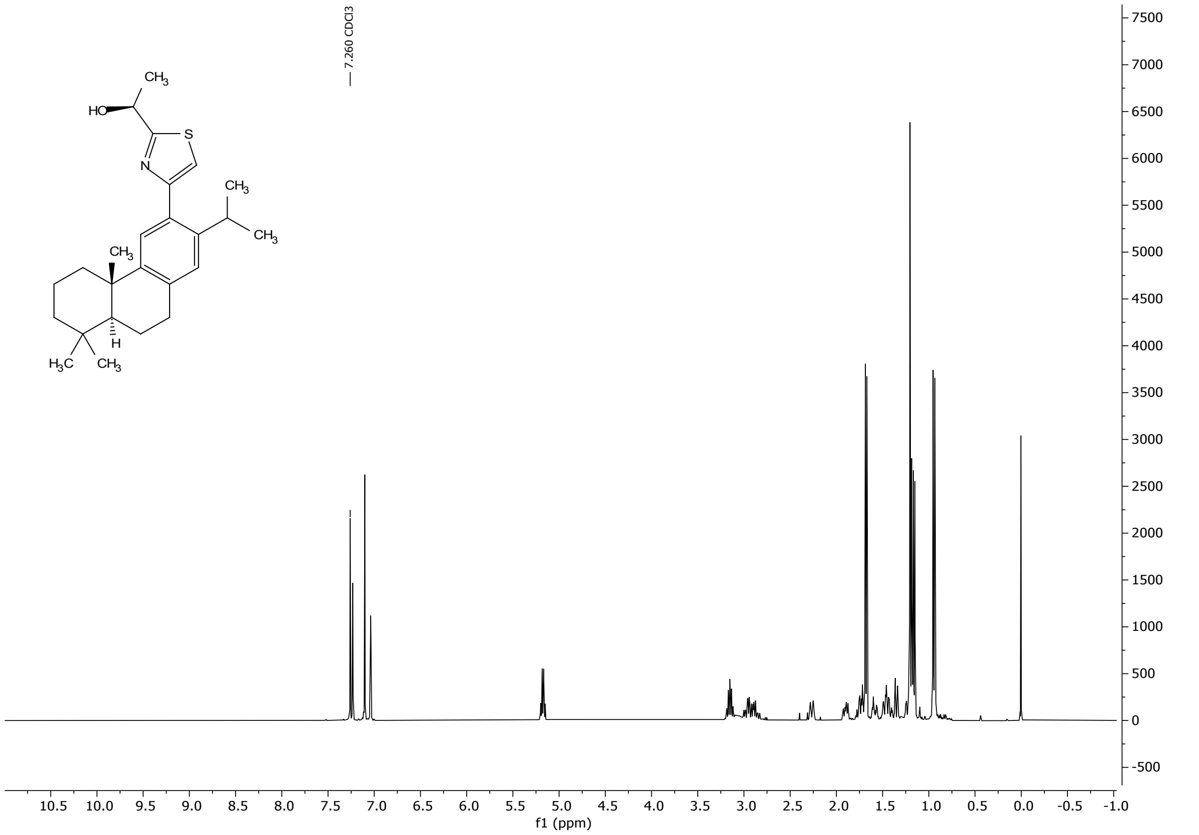

^{1}H NMR of compound **30** in $CDCl_3$

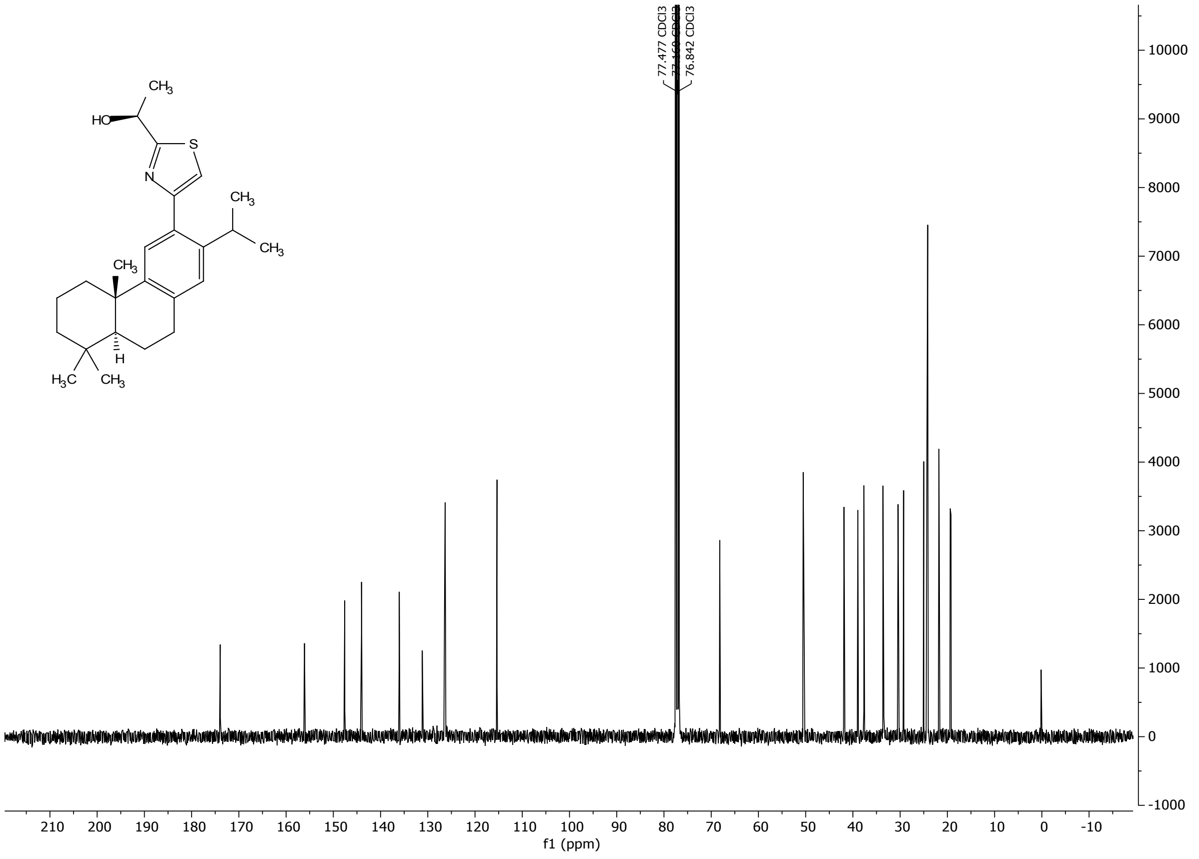

^{13}C NMR of compound **30** in $CDCl_3$

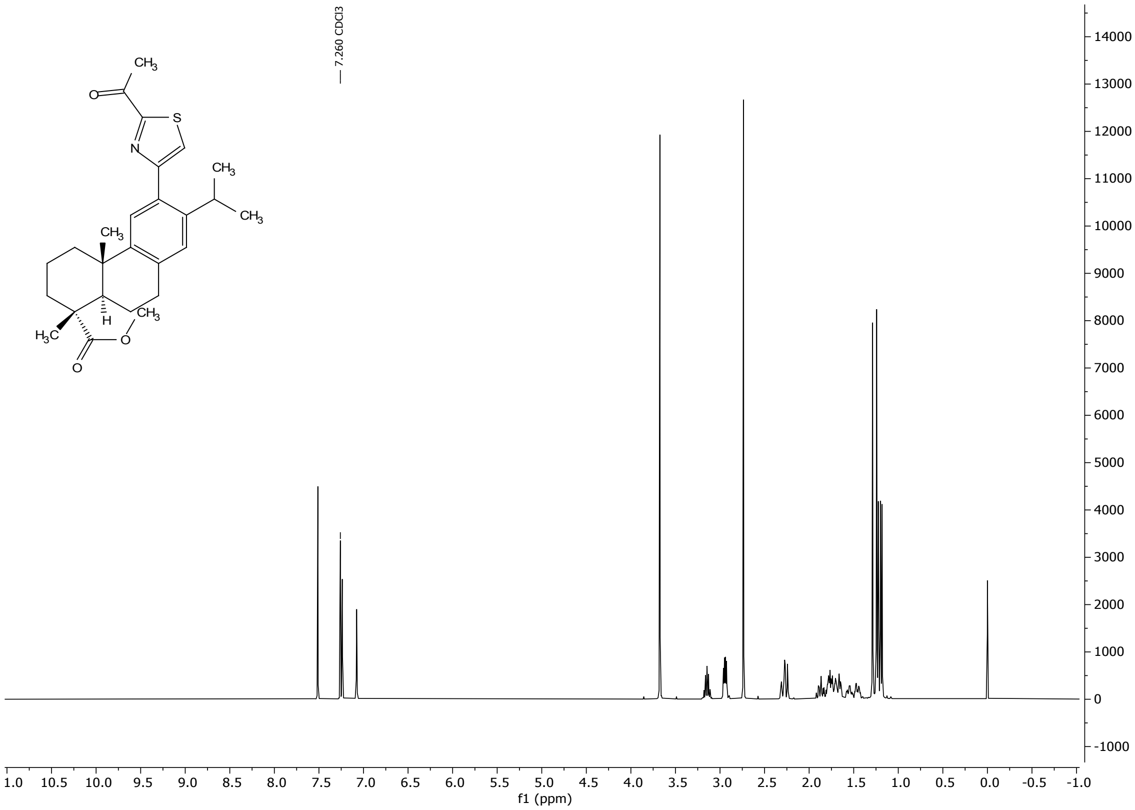

^{1}H NMR of compound **31** in $CDCl_3$

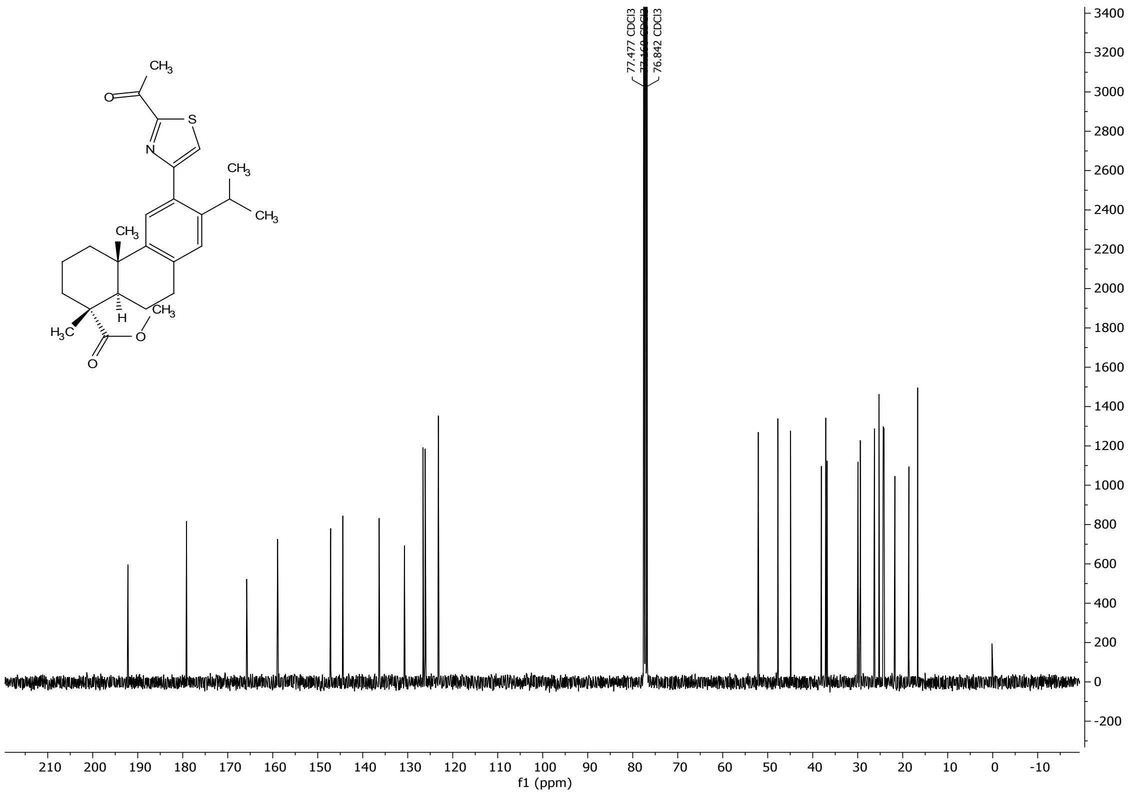

^{13}C NMR of compound **31** in $CDCl_3$

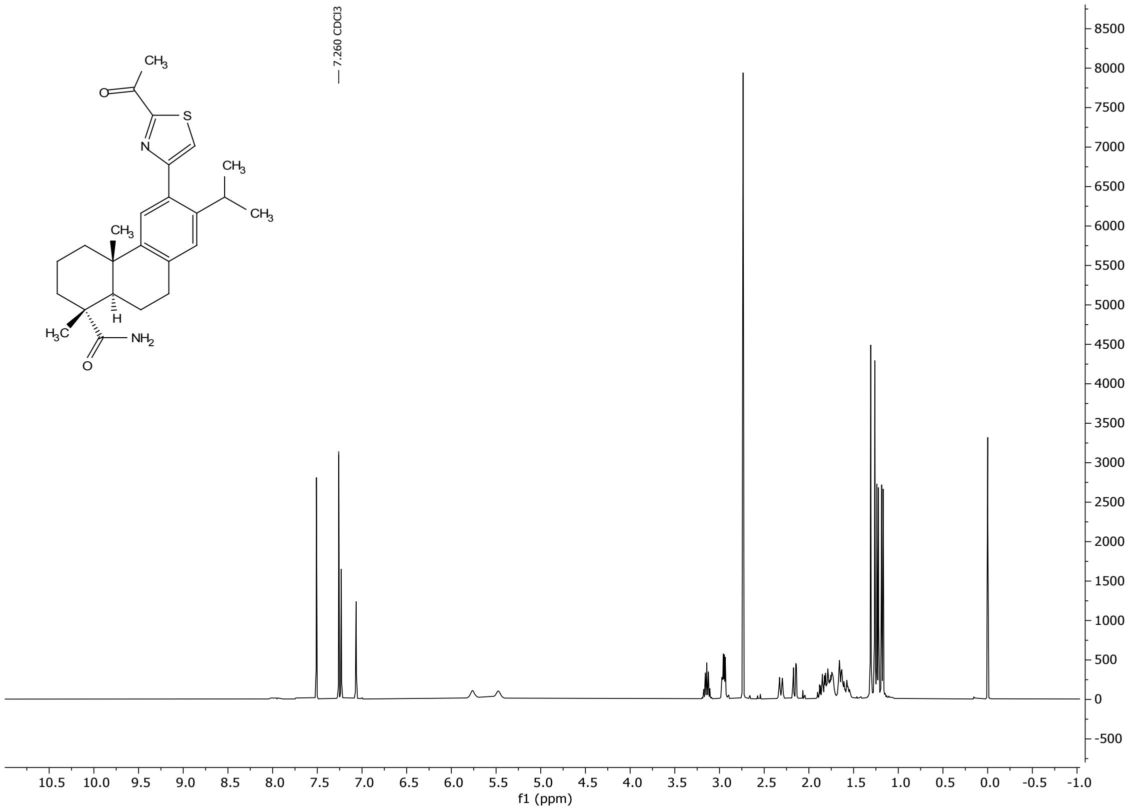

^{1}H NMR of compound **32** in $CDCl_3$

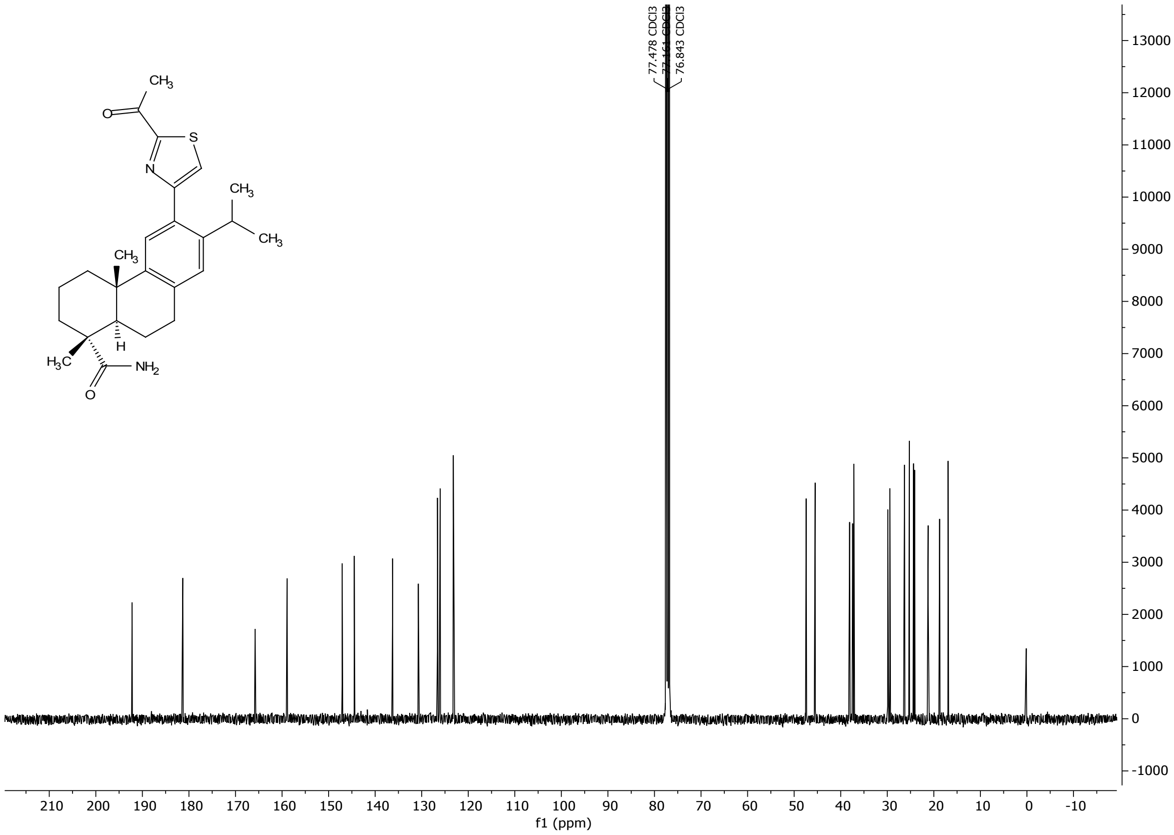

^{13}C NMR of compound **32** in $CDCl_3$

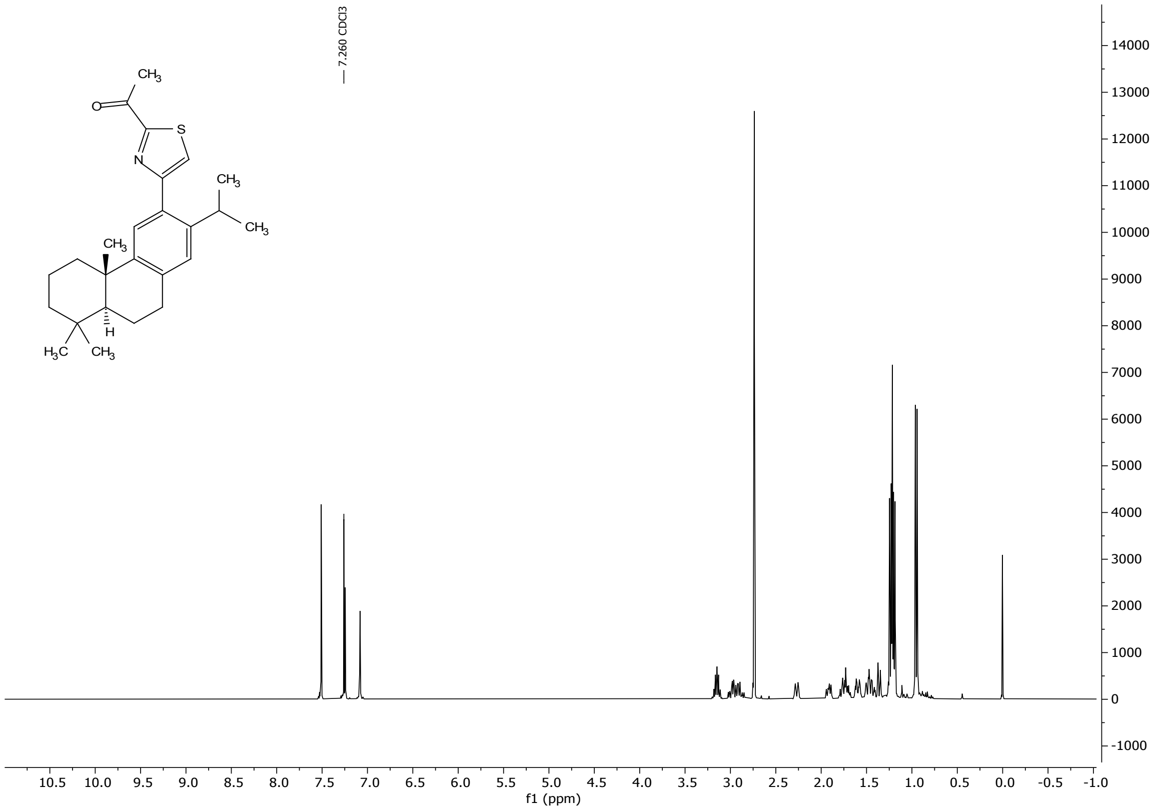

^{1}H NMR of compound **33** in $CDCl_3$

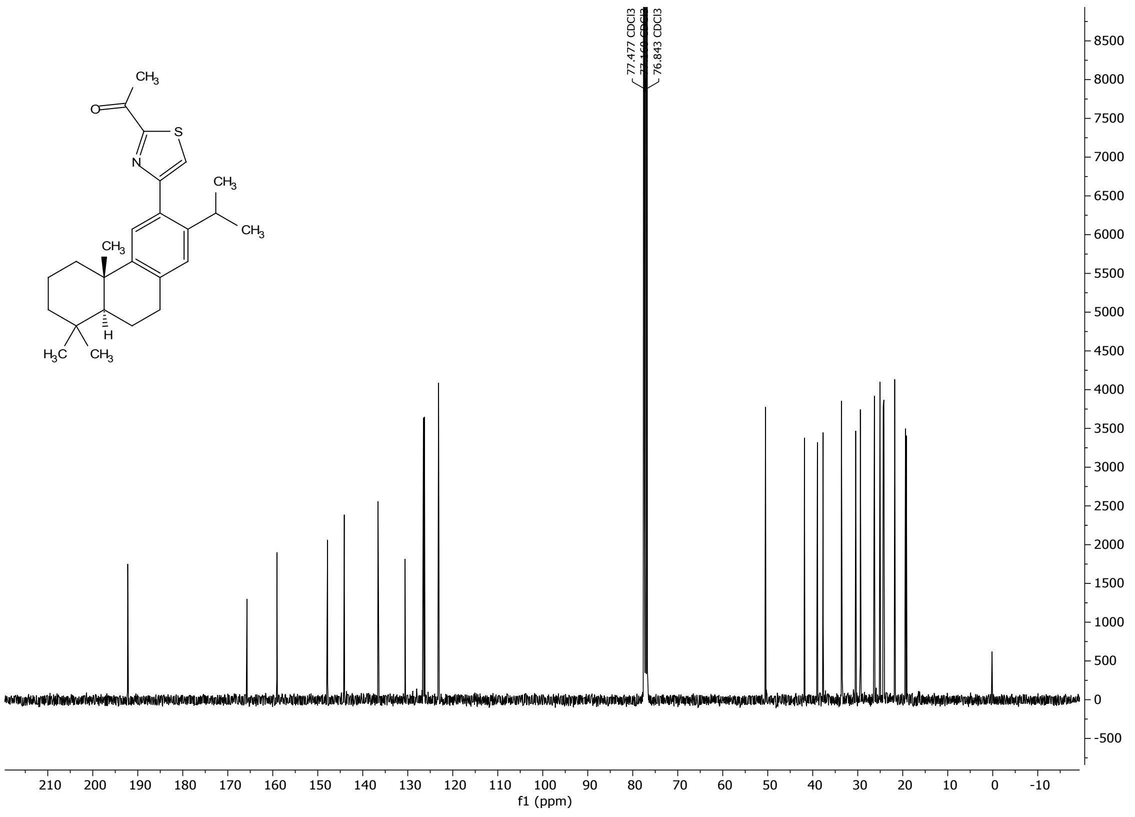

^{13}C NMR of compound **33** in $CDCl_3$

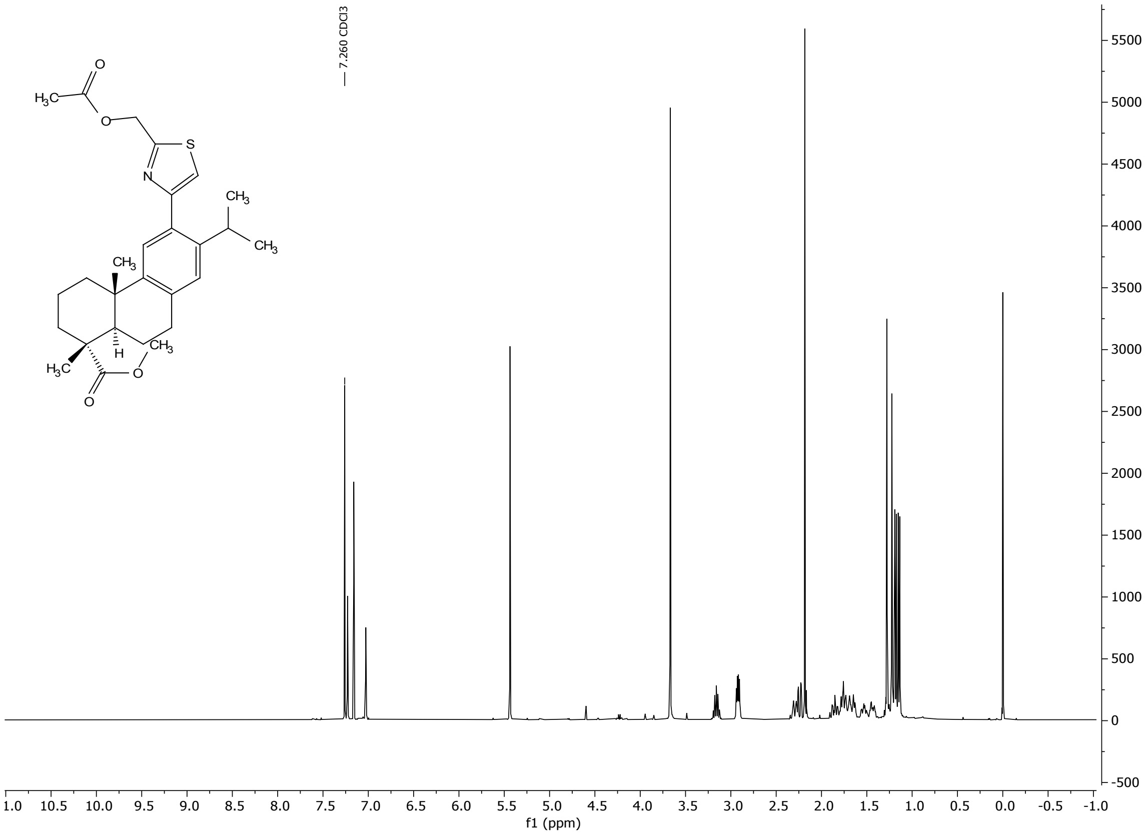

^{1}H NMR of compound **34** in $CDCl_3$

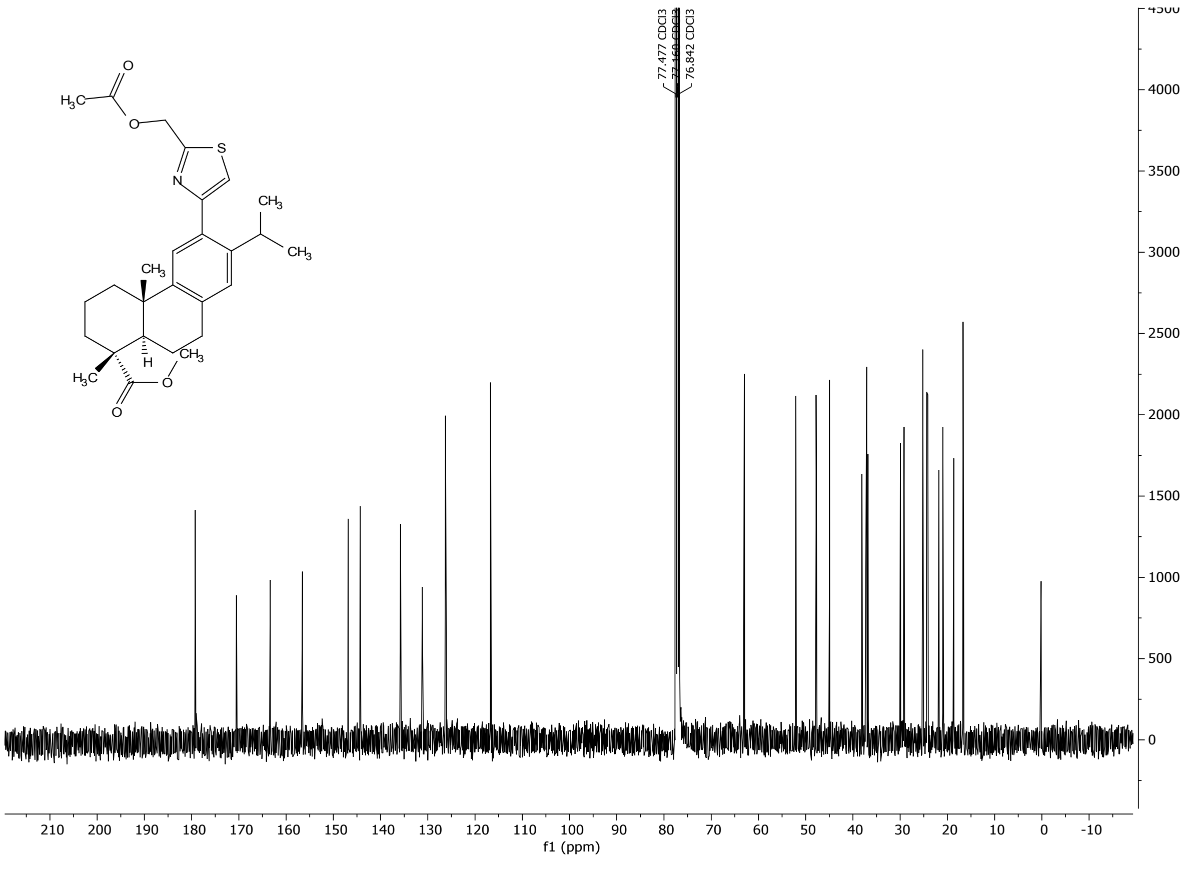

^{13}C NMR of compound **34** in $CDCl_3$

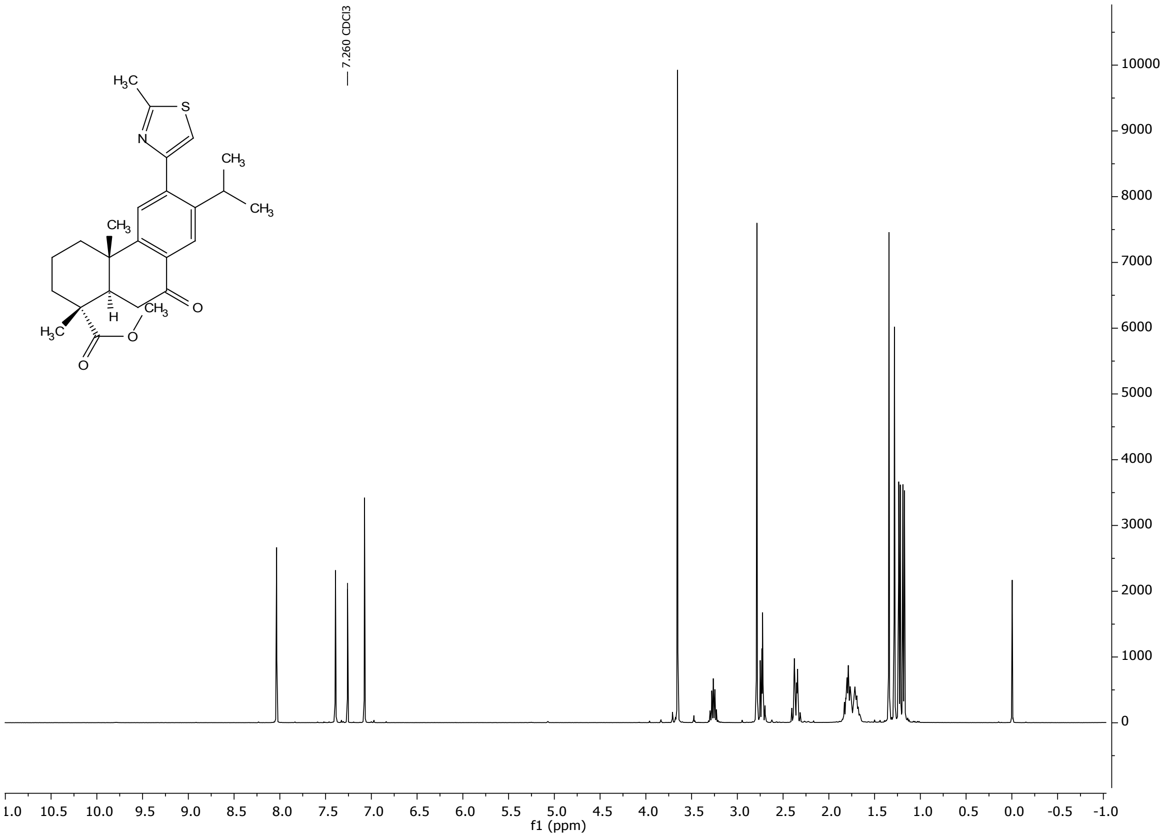

^{1}H NMR of compound **35** in $CDCl_3$

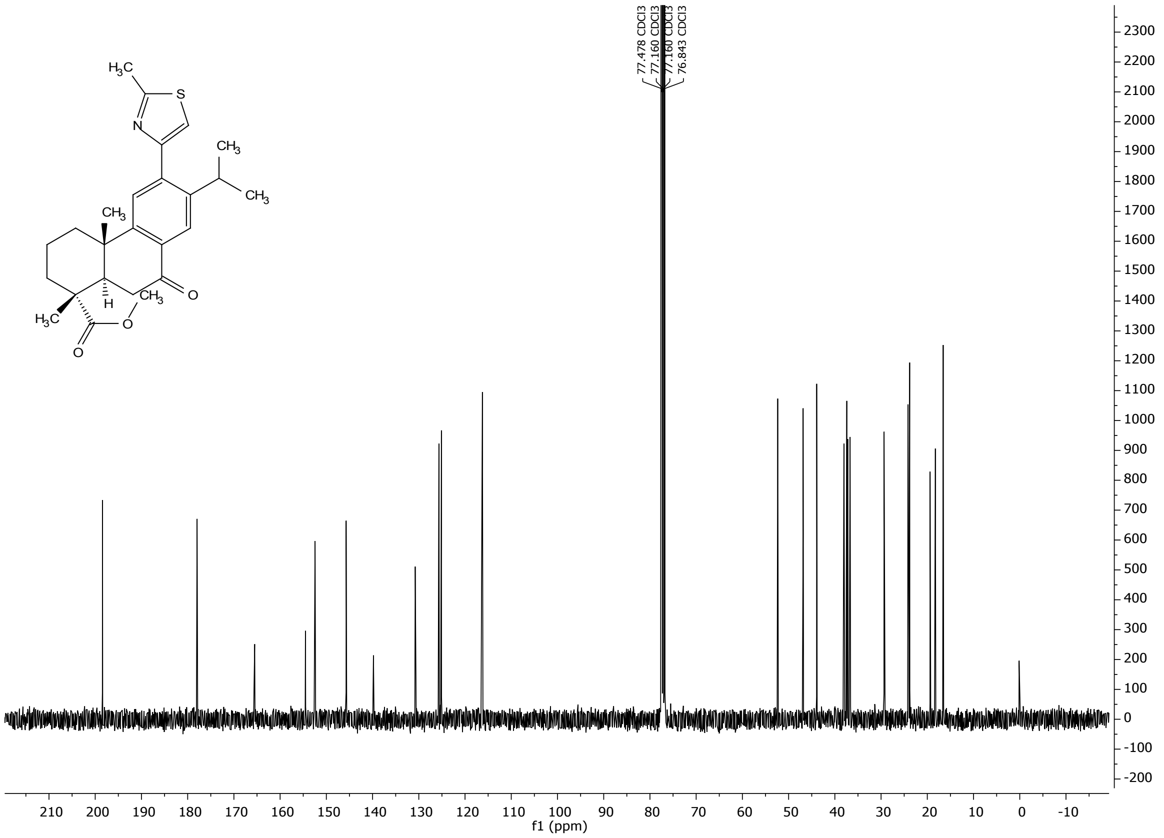

^{13}C NMR of compound **35** in $CDCl_3$

7. References

(1) Zhou, J.; Liu, P.; Chen, S.; Wu, Y.; Wang, D.; Jia, Z.; Qiao, L.; Frietze, W.; Xia, M.; Dai, Y. Processes of Preparing a JAK1 Inhibitor and New Forms Thereto. WO2015/168246, 2015.

(2) Schmidt, U.; Gleich, P.; Griesser, H.; Utz, R. Amino Acids and Peptides; 58 Synthesis of Optically Active 2-(1-Hydroxyalkyl)-Thiazole-4-Carboxylic Acids and 2-(1-Aminoalkyl)-Thiazole-4-Carboxylic Acids. *Synthesis (Stuttg).* **1986**, *12*, 992–998. https://doi.org/10.1055/s-1986-31847.

(3) Fukata, M.; Fukata, Y.; Adesnik, H.; Nicoll, R. A.; Bredt, D. S. Identification of PSD-95 Palmitoylating Enzymes. *Neuron* **2004**, *44* (6), 987–996. https://doi.org/https://doi.org/10.1016/j.neuron.2004.12.005.

(4) Longo, P. A.; Kavran, J. M.; Kim, M.-S.; Leahy, D. J. Chapter Eighteen - Transient Mammalian Cell Transfection with Polyethylenimine (PEI). In *Laboratory Methods in Enzymology: DNA*; Lorsch, J. B. T.-M. in E., Ed.; Academic Press, 2013; Vol. 529, pp 227–240. https://doi.org/10.1016/B978-0-12-418687-3.00018-5.

(5) Greaves, J.; Tomkinson, N. C. O. Detection of Heterogeneous Protein S-Acylation in Cells BT - Protein Lipidation: Methods and Protocols; Linder, M. E., Ed.; Springer New York: New York, NY, 2019; pp 13–33. https://doi.org/10.1007/978-1-4939-9532-5_2.

(6) Morris, G. M.; Huey, R.; Lindstrom, W.; Sanner, M. F.; Belew, R. K.; Goodsell, D. S.; Olson, A. J. AutoDock4 and AutoDockTools4: Automated Docking with Selective Receptor Flexibility. *J. Comput. Chem.* **2009**, *30* (16), 2785–2791. https://doi.org/https://doi.org/10.1002/jcc.21256.

(7) Jumper, J.; Evans, R.; Pritzel, A.; Green, T.; Figurnov, M.; Ronneberger, O.; Tunyasuvunakool, K.; Bates, R.; Žídek, A.; Potapenko, A.; Bridgland, A.; Meyer, C.; Kohl, S. A. A.; Ballard, A. J.; Cowie, A.; Romera-Paredes, B.; Nikolov, S.; Jain, R.; Adler, J.; Back, T.; Petersen, S.; Reiman, D.; Clancy, E.; Zielinski, M.; Steinegger, M.; Pacholska, M.; Berghammer, T.; Bodenstein, S.; Silver, D.; Vinyals, O.; Senior, A. W.; Kavukcuoglu, K.; Kohli, P.; Hassabis, D. Highly Accurate Protein Structure Prediction with AlphaFold. *Nature* **2021**, *596* (7873), 583–589. https://doi.org/10.1038/s41586-021-03819-2.

(8) Vainio, M. J.; Johnson, M. S. Generating Conformer Ensembles Using a Multiobjective Genetic Algorithm. *J. Chem. Inf. Model.* **2007**, *47* (6), 2462–2474. https://doi.org/10.1021/ci6005646.

(9) Ahonen, T. J.; Savinainen, J. R.; Yli-Kauhaluoma, J. T.; Kalso, E. A.; Laitinen, J. T.; Moreira, V. M. Discovery of 12-Thiazole Abietanes as Selective Inhibitors of the Human Metabolic Serine Hydrolase HABHD16A. *ACS Med. Chem. Lett.* **2018**, *9* (12), 1269–1273. https://doi.org/10.1021/acsmedchemlett.8b00442.
